# Hydrophobic, Acid-Free Zeolite-Confined Pt–Cu Nanoalloys Break Activity–Selectivity Limits in Low-Temperature Methane-to-Methanol Oxidation

**DOI:** 10.1021/jacs.5c07414

**Published:** 2025-08-11

**Authors:** Akira Oda, Koyo Ichino, Yuta Yamamoto, Takeshi Ohtsu, Wei Shi, Yoshiharu Sawada, Jun Kumagai, Kyoichi Sawabe, Atsushi Satsuma

**Affiliations:** † Department of Materials Chemistry, Graduate School of Engineering, 12965 Nagoya University, Nagoya 464-8603, Japan; ‡ Institute for Catalysis, Hokkaido University, Sapporo 001-0021, Japan; § Institute of Materials and Systems for Sustainability, Nagoya University, Nagoya 464-8603, Japan; ∥ Technical Center, Nagoya University, Nagoya, Aichi 464-8601, Japan

## Abstract

The direct oxidation of methane (CH_4_) to methanol (CH_3_OH) remains a formidable challenge due to the inertness of CH_4_ and the tendency of CH_3_OH to overoxidize. Here, we report Pt–Cu nanoalloys encapsulated within hydrophobic, acid-free silicalite-1 (S-1) zeolite that breaks activity–selectivity limits in CH_4_ oxidation to CH_3_OH. The best catalyst exhibits a CH_3_OH productivity of 134 mol of CH_3_OH per mol of Pt per hour and a selectivity of 95% at 150 °C. Kinetic and spectroscopic studies revealed a sequential oxidation mechanism: CH_4_ is first oxidized to methyl hydroperoxide (CH_3_OOH) by in situ generated hydrogen peroxide, which subsequently converts to CH_3_OH. The catalytic reaction proceeds with an apparent activation energy of only 42 kJ/mol, the lowest reported to date. The outstanding performance arises from the synergy of the Pt–Cu alloy sites and the hydrophobic pore of S-1. Pt–Cu alloy sites specifically generate oxidizing species and selectively form CH_3_OH, which was not achieved by single metal catalysts and other bimetallic catalysts. A confined hydrophobic, acid-free environment enables rapid extraction of the CH_3_OH from the reaction field and thereby prevents overoxidation. These findings highlight how precious control over both the composition and the local environment of Pt–Cu nanoalloys can markedly enhance the catalytic oxidation of CH_4_ to CH_3_OH.

## Graphical abstract

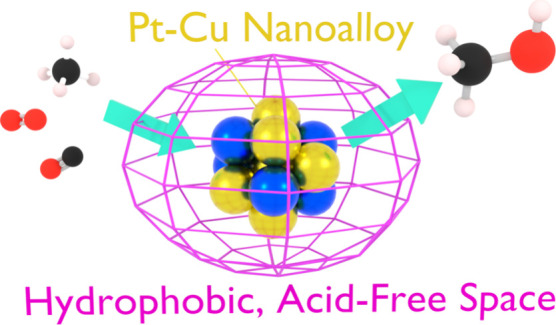

## Introduction

Methane (CH_4_), the main component of natural gas, is an abundant and inexpensive feedstock with significant potential for sustainable energy and chemical conversion.^1,2^ Among the various transformation pathways, the selective oxidation of CH_4_ to methanol (CH_3_OH) is particularly attractive, as CH_3_OH serves as a versatile platform molecule for fuels and chemical feedstock.^3^ However, commercial CH_3_OH production still relies on a two-step process involving energy-intensive steam reforming followed by catalytic CH_3_OH synthesis, which requires high temperatures and large-scale infrastructure. Direct conversion of CH_4_ to CH_3_OH remains highly challenging due to the strong C–H bond of CH_4_ (∼435 kJ/mol) and the inherent instability of CH_3_OH under oxidative conditions, often leading to overoxidation to undesirable byproducts such as formic acid (HCOOH) and CO_2_. Various catalytic systems, including metal-containing zeolites, have been explored using oxidants such as hydrogen peroxide (H_2_O_2_),^4,5^ oxygen (O_2_),^6–8^ water (H_2_O),^9,10^ or nitrous oxide,^11,12^ yet achieving both high CH_3_OH selectivity and productivity remains elusive.

In the presence of a biological reductant, typically dihydronicotinamide adenine dinucleotide, the reductive activation of O_2_ is a general strategy employed by enzymatic systems such as methane monooxygenase to achieve selective oxidation.^13^ Inspired by these biological processes, a CO-assisted mechanism for selective CH_4_ oxidation on heterogeneous catalysts has recently attracted considerable attention.^14^ By utilizing CO as a reductant in aqueous media, O_2_ activation is promoted, leading to the in situ generation of H_2_O_2_ and hydroxyl radicals (OH^•^).^15–18^ Using these reactive oxygen species as the oxidants, the active metal sites catalytically oxidize CH_4_ to organic oxygenates while facilitating the rapid release of the oxygenates into the water solvent. A wide range of heterogeneous catalysts containing single atoms or nanoparticles of noble metals have been reported for the CO-assisted oxidation of CH_4_. These include Rh,^14,17,19–24^ Ru,^25^ Ir,^15,26,27^ Pd,^28^ Au,^16,29,30^ and Pt^31^-based catalysts. To enhance the CH_3_OH selectivity, several key strategies have been identified. These include choosing an appropriate active metal,^15,25^ controlling the metal particle size and loading,^16,21,31^ tailoring the local structure or environment,^20,26^ adding a second metal complex (e.g., CuCl_2_) as a homogeneous catalyst,^32,33^ removing Brønsted acid sites from the catalyst surface,^14,34^ and inducing hydrophobicity through surface modification.^35,36^ Nevertheless, achieving a high CH_3_OH productivity while maintaining high selectivity remains challenging; existing heterogeneous catalysts cannot perform better than a CH_3_OH productivity of 73 mol of CH_3_OH per mol of noble metal (NM) per hour (Table S1). To overcome this limitation, coaddition of an excess amount of Cu-based homogeneous catalysts is needed.^33^

Here, we report that precisely designing a nanoalloy catalyst confined within a solid acid-free hydrophobic reaction space allows us to break through the existing activity–selectivity limits in the CO-assisted oxidation of CH_4_ to CH_3_OH. We focus on Pt–Cu alloy nanoparticles encapsulated within the hydrophobic micropores of silicalite-1 zeolite (PtCu@S-1). Pt–Cu alloys are well-known for their high activity in C–H oxidation,^37^ and a recent study has demonstrated that Pt is a particularly useful element in CO-assisted oxidation mechanisms.^18^ Furthermore, alloying Pt with Cu, which can stabilize methoxy intermediates,^38^ is expected to further enhance CH_3_OH selectivity. Silicalite-1 is a pure silica zeolite having a hydrophobic nature and exhibits a relatively high transport efficiency for CH_3_OH compared to water.^39^ Therefore, using the hydrophobic micropores of S-1 as the reaction nanospace, CH_3_OH, once synthesized by the active sites, can be quickly expelled from the micropores, reducing the side reaction rate and thereby enhancing CH_3_OH selectivity. An additional advantage of S-1 is its lack of Brønsted acid sites, which effectively suppresses acid-catalyzed pathways responsible for the formation of undesirable byproducts such as HCOOH and CH_3_COOH.^14,23,34^ Taken together, these insights suggest that PtCu@S-1 could overcome the activity–selectivity limits faced by earlier systems. Although the precise encapsulation of alloy nanoparticles inside micropores of S-1 has long been challenging, recent advances in one-step hydrothermal synthesis have made this approach feasible.^40–43^ Despite these advances, its potential for selective CH_4_ oxidation has not been explored.

The purpose of this study is to demonstrate the significance of encapsulating specific alloy nanoparticles within a solid acid-free hydrophobic nanospace. First, a series of model catalysts are designed and characterized, including (1) Pt–Cu alloy nanoparticles with varying Pt/Cu molar ratios encapsulated in S-1, (2) Pt–Cu alloy nanoparticles encapsulated in MFI zeolites with different Si/Al molar ratios (to introduce varying amounts of Brønsted acid sites), and (3) Pt–Cu alloy nanoparticles supported on the external surface of S-1. Next, the catalytic activity and selectivity of these materials in CO-assisted oxidation of CH_4_ are evaluated. Based on the obtained data, structure–function relationships are clarified. Finally, through controlled kinetic and spectroscopic experiments using the best catalyst, the reaction mechanism is investigated. By comparison of the performance of PtCu@S-1 with that of analogous Pt-only or Cu-only catalysts, the synergistic interplay between Pt and Cu sites within the micropores of S-1 is clarified.

## Results and Discussion

### Synthesis and Characterization

Pt–Cu alloy nanoparticles encapsulated in S-1 (PtCu@S-1) were synthesized via a one-step hydrothermal method, as illustrated in Figure 1A.^40–43^ Briefly, a zeolite synthesis gel containing ethylenediamine complexes of Pt and Cu was subjected to static hydrothermal treatment at 170 °C for 3 days to crystallize the zeolite. The resulting material was then calcined under flowing H_2_ at 500 °C to form the alloy within the micropores and subsequently decompose organic structure-directing agents, tetrapropylammonium cations (TPA^+^) that occupy the zeolite channels. Details of the synthesis procedure are provided in the Supporting Information (Materials and Methods section and Table S2). Simultaneous thermogravimetry and differential thermal analysis, as well as textural properties determined by N_2_-adsorption isotherms, confirmed complete removal of TPA^+^ (Figure S1 and Table S3), in agreement with the relevant literature,^40,42^ and the liberated, metal-encapsulated micropores are accessible for catalysis. The calcination process did not change the concentration of defects (Si–OH) influencing the hydrophobicity of S-1 zeolite, as evidenced by ^29^Si magic-angle spinning nuclear magnetic resonance spectra (Figure S2). The catalyst synthesized with a Pt:Cu molar ratio of 1:1 is denoted as Pt1Cu1@S-1. For comparison, a Pt-only encapsulated catalyst (Pt@S-1) and a Cu-only encapsulated catalyst (Cu@S-1) were synthesized using the same one-step method but with only the Pt or Cu ethylenediamine complex, respectively. Additionally, an S-1 supported Pt–Cu nanoalloy catalyst was also prepared by impregnating S-1 with Pt and Cu (1:1) followed by reduction. This externally supported catalyst is denoted as Pt1Cu1/S-1. The chemical compositions of mother gels and resultant catalysts determined by inductively coupled plasma–optical emission spectroscopy are summarized in Tables S2 and S3.

**Figure 1:**
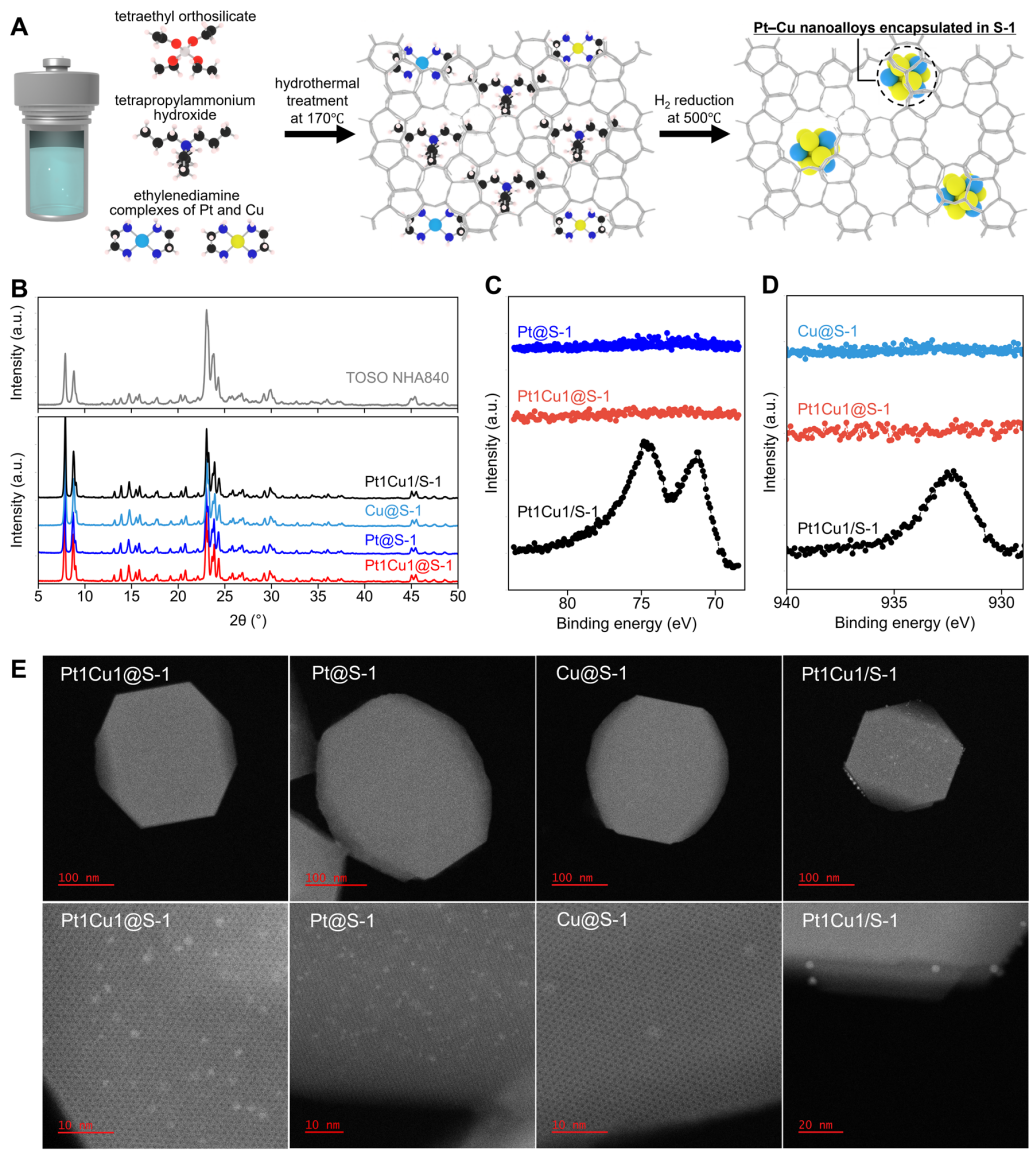
(A) Schematic representation of the one-step hydrothermal synthesis used to encapsulate Pt–Cu alloy nanoparticles within the S-1 framework. Legend: black, C; blue, N; red, O; light blue, Cu; yellow, Pt; white, H; gray frame, MFI-type zeolite. (B) XRD profiles. (C, D) XPS of Pt 4f and Cu 2p regions. (E) Representative HAADF-STEM images of each catalyst.

Powder X-ray diffraction (XRD) patterns (Figure 1B) confirmed that all catalysts exhibit the characteristic XRD patterns of the MFI-type zeolite, indicating the successful crystallization of the MFI-type zeolite. No diffraction lines corresponding to Pt or Cu metal phases were observed for any of the catalysts, suggesting that metal species were highly dispersed. To assess the distribution of Pt and Cu, X-ray photoelectron spectroscopy (XPS) was performed on these catalysts (Figure 1C,D). XPS, being surface-sensitive (probe depth of ∼3 nm), readily detects metal species located on the external surface of the zeolite.^40,42,44^ Actually, the impregnated Pt1Cu1/S-1 catalyst provided clear Pt 4f and Cu 2p signals. In contrast, the one-step hydrothermally synthesized catalysts (Pt1Cu1@S-1, Pt@S-1, and Cu@S-1) exhibited negligible signals; therefore, the fraction of metals located on the external surface of S-1 is extremely low, suggesting that in these catalysts, the Pt and Cu species are predominantly encapsulated within the zeolite micropores.

High-angle annular dark-field scanning transmission electron microscopy (HAADF-STEM) provided direct evidence of the metal nanoparticle locations (Figure 1E). Additional HAADF-STEM images are available in the Supporting Information (Figures S3–S6). In the Pt1Cu1/S-1 catalyst, abundant 2–5 nm metal nanoparticles were observed on the external surface of S-1, consistent with the XPS results. Lattice fringes corresponding to Pt–Cu alloy nanocrystals were also visible (see Figure S3 in the Supporting Information), confirming that Pt and Cu form an alloy. In contrast, the catalysts prepared by the one-step hydrothermal synthesis method (PtCu@S-1, Pt@S-1, and Cu@S-1) showed virtually no metal nanoparticles on the external surface of S-1; instead, uniformly dispersed nanoparticles were observed within the zeolite micropores. These observations, together with the XPS data, demonstrate that the one-step hydrothermal synthesis method effectively encapsulates Pt and Cu within the S-1 micropores. The average particle sizes were 1.2 ± 0.4 nm for Pt1Cu1@S-1, 0.9 ± 0.4 nm for Pt@S-1, 2.0 ± 0.8 nm for Cu@S-1, and 3.0 ± 1.3 nm for Pt1Cu1/S-1 (Figure S7).

X-ray absorption fine structure (XAFS) spectroscopy was used to probe the local atomic structure and electronic state of Pt and Cu in the encapsulated Pt–Cu alloys. Figure 2A shows the Pt L_III_-edge X-ray absorption near-edge structure (XANES) spectra. The white-line intensity for Pt1Cu1@S-1 was lower than that for Pt@S-1 and was close to that of a metallic Pt foil, suggesting that Pt exists in a reduced form as a result of interaction with Cu. In comparison, Pt@S-1 exhibited a white-line intensity that was higher than that of Pt1Cu1@S-1, indicating that Pt is present predominantly in a more oxidized state. It is expected that the absence of Cu allowed Pt atoms to interact more strongly with the zeolite framework oxygen, thereby stabilizing partially oxidized Pt species. Figure 2B displays the Cu K-edge XANES spectra. Compared to Cu@S-1, the absorption edge of Pt1Cu1@S-1 was shifted to lower energy, indicating that Cu is also in a lower oxidation state (more reduced) due to alloying with Pt. Moreover, the XANES spectrum of Pt1Cu1@S-1 could not be reproduced by a linear combination of reference spectra for Cu@S-1, Cu foil, Cu_2_O, and CuO (Figure S8), implying the formation of a Cu species having a unique local environment. The spectral feature observed for Pt1Cu1@S-1 is consistent with that reported for Pt–Cu alloy nanoparticles,^45,46^ thereby supporting the formation of Pt–Cu nanoalloys within the micropores of S-1. Focusing on Cu@S-1, the absorption edge was shifted toward higher energy relative to Cu foil and appears closer to that of Cu_2_O. This suggests that, because the Cu species interact directly with the zeolite framework oxygen rather than with Pt, more oxidized Cu species predominated compared with those in Pt1Cu1@S-1.

**Figure 2:**
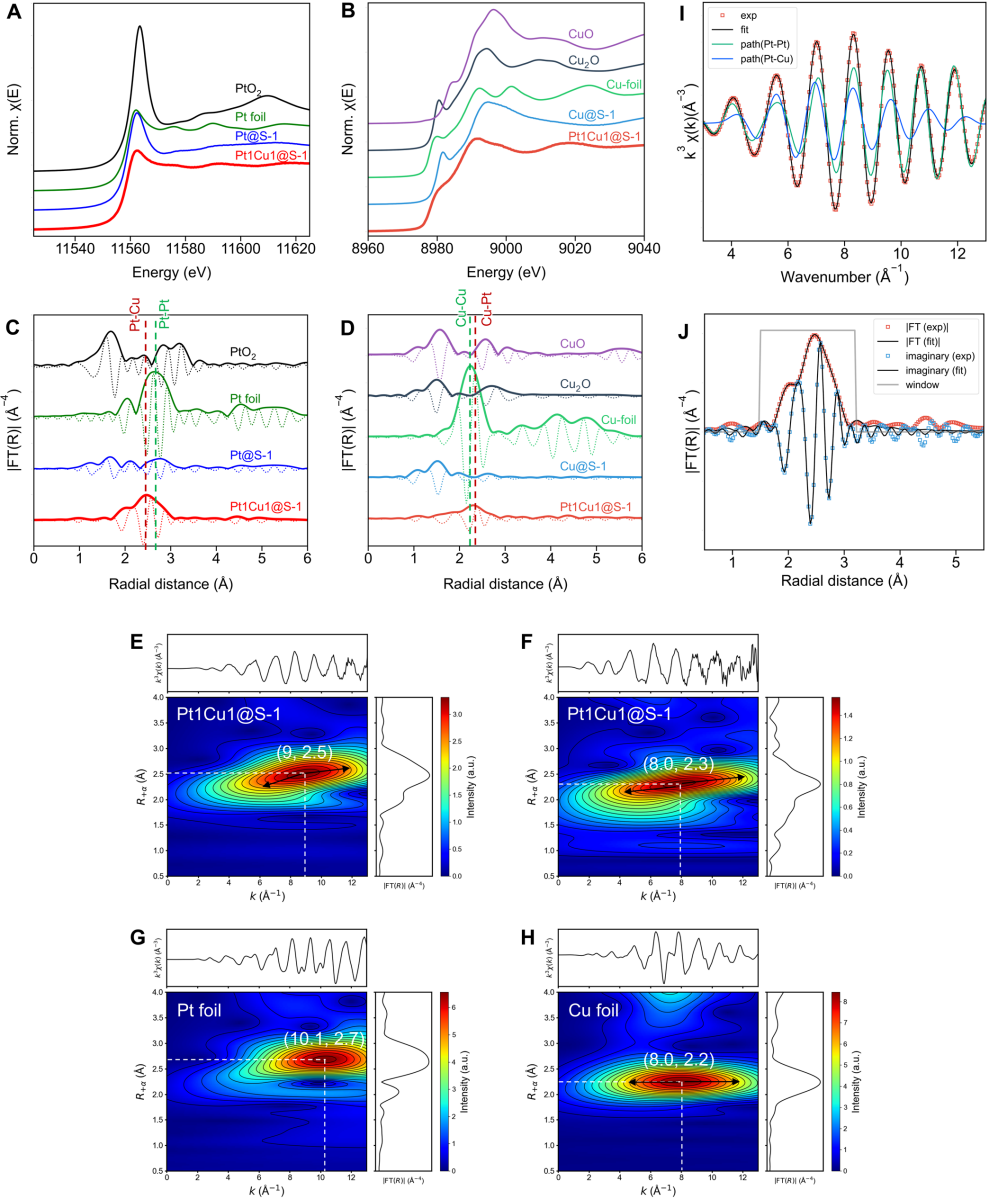
(A, B) Pt L_III_-edge and Cu K-edge XANES spectra. (C, D) Pt L_III_-edge and Cu K-edge FT-EXAFS spectra. (E) Pt L_III_ and (F) Cu K-edge WT-EXAFS of Pt1Cu1@S-1. Reference data: (G) Pt foil and (H) Cu foil. (I) Curve-fitting of the Pt L_III_-edge EXAFS function for Pt1Cu1@S-1 assuming two contributions: Pt–Cu and Pt–Pt backscattering. (J) Inverse FT of the best fit EXAFS function with the experimental FT-EXAFS spectrum.

The extended X-ray absorption fine structure (EXAFS) was analyzed to further confirm the Pt–Cu alloys. The Fourier transform EXAFS (FT-EXAFS) spectra for the Pt L_III_-edge and Cu K-edge are shown in Figure 2C,D. Pt1Cu1@S-1 exhibits a prominent backscattering peak at a radial distance shorter than the Pt–Pt distance in Pt foil but longer than the Cu–Cu distance in Cu foil. This observation supports the presence of a Pt–Cu bond with a distance intermediate between those of Pt–Pt and Cu–Cu. This assignment is further supported by wavelet-transformed EXAFS (WT-EXAFS) as shown in Figure 2E–H. At the Pt L_III_-edge of Pt1Cu1@S-1, a lobe-shaped feature was observed that cannot be explained solely by Pt–Pt backscattering, in contrast to the Pt foil. This is attributed not only to Pt–Pt backscattering but also to Pt–Cu backscattering, which contributes to the lobe localized in the low-*k* (∼6 Å) and low-*R* (∼2.1 Å) regions. Similarly, at the Cu K-edge, a complex spectral shape was observed that cannot be explained by Cu–Cu backscattering in Cu foil alone. While lobes with maxima of WT coefficient appear at (*k*, *R*) positions similar to those of Cu–Cu backscattering, the lobes extend into the high-*R* (>2.3 Å) region in the high-*k* range (>8 Å^–1^). This clearly indicates the involvement of heavy-element-derived backscattering, specifically Cu–Pt backscattering. To quantify the local structure, EXAFS curve-fitting was performed (Figure 2I,J). The fitting confirmed that two scattering paths (Pt–Cu and Pt–Pt) are necessary to reproduce the Pt1Cu1@S-1 data, further validating the alloy nature. The best fit bond lengths were 2.64 Å for Pt–Cu and 2.71 Å for Pt–Pt (see Table S4), in agreement with literature values for Pt–Cu nanoalloys,^37,47^ corroborating that Pt–Cu alloy nanoparticles are formed inside the S-1 micropores. In contrast, prominent Pt–O and Cu–O backscattering features were observed for Pt@S-1 and Cu@S-1, respectively, suggesting strong interactions between the metals and lattice oxygens.

CO adsorption Fourier transform infrared spectroscopy (CO-FTIR) was employed to analyze the surface sites of the encapsulated Pt–Cu alloy nanoparticles and their monometallic analogues. Since the position, intensity, and full width at half-maximum (fwhm) of the ν_CO_ bands for adsorbed CO species reflect the nature of the active sites on the catalyst surface, this technique provides detailed structural information about the active sites.^18,40,42,44,48,49^ After CO adsorption, the CO gas supply was turned off, and the system was purged with Ar. Time-resolved measurements were carried out during the purge process to monitor both irreversibly and reversibly adsorbed CO on the catalyst surface (Figure 3A–D). For Pt@S-1, the ν_CO_ band (band I) was observed at 2063–2060 cm^–1^, characteristic of CO adsorbed on atop Pt sites; this band shifted slightly to lower energy during the Ar purge. For Pt1Cu1@S-1, band I was observed at 2049–2044 cm^–1^, which was 14–16 cm^–1^ lower than that observed in Pt@S-1. This energy difference shows that Pt sites in the Pt–Cu alloy are more electron-rich than in the Pt-only catalyst. The excess charge originates from electron donation by neighboring Cu atoms, whose lower electronegativity drives electron transfer to Pt and, in turn, weakens the C–O bond of the CO species adsorbed on the Pt sites. Similar effects have been reported in previous studies of Pt–Cu alloy catalysts.^43,48,50^ Additionally, the ν_CO_ band (band II) was transiently detected at 2116 cm^–1^ during the Ar purge; this band II, which can be assigned to the CO species adsorbed on atop Cu sites, diminished during the purge process. Notably, as band II (Cu–CO) decayed, band I (Pt–CO) grew in intensity, shifted further to lower energy, and became sharper (narrower fwhm). The variations in the band intensities correlated (Figure 3E,F), indicating that bands I and II are related to the Pt and Cu sites in close proximity; a bimetallic ensemble site was further evidenced. In contrast, for the supported catalyst (Pt1Cu1/S-1), qualitatively similar bands I and II were observed but with much lower intensity because of a lower surface area (i.e., larger particles). The time-dependent behavior of band I in PtCu/S-1 also differed from that in Pt1Cu1@S-1, suggesting that the geometries of Pt–Cu alloy sites are different. On the other hand, Cu@S-1 exhibited only a very weak band II before purging, suggesting that Cu nanoparticles bind CO weakly.

**Figure 3:**
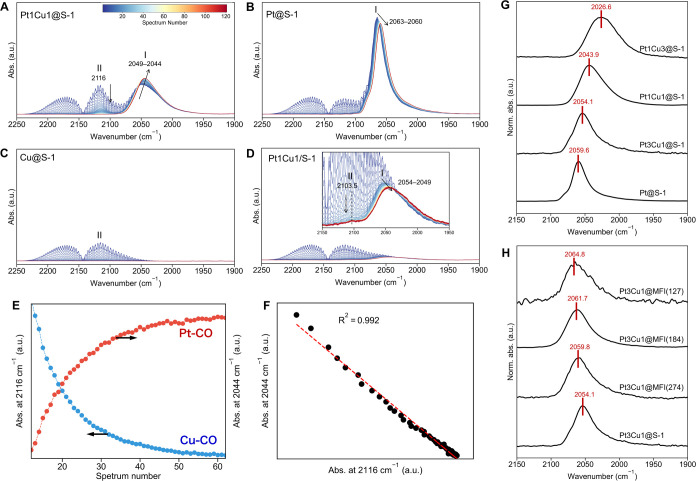
(A–D) Time-resolved CO-FTIR spectra under Ar purge following CO adsorption. The characteristics of the change in band intensity and position over time are indicated by arrows. (E) Time course of intensities for ν_CO_ bands at 2116 and 2044 cm^–1^. To eliminate the contribution of the signals of gas-phase CO, a scan range (12–60 spectrum numbers) where almost all gas-phase CO is removed from the system is shown. (F) Correlation plot between intensities of ν_CO_ bands at 2116 and 2044 cm^–1^. (G, H) CO-FTIR spectra of PtCu@S-1 with different Cu/Pt molar ratios and Pt3Cu1@MFI with different Si/Al molar ratios. These spectra were collected after CO adsorption and subsequent Ar purge. For comparison, the absorbance of the spectra was normalized to the highest intensity.

Having established the structural characteristics of the Pt–Cu encapsulated catalysts, variants were synthesized to probe the impact of the alloy composition and framework aluminum (acid sites). In short, PtCu@S-1 catalysts with higher or lower Cu content (denoted Pt3Cu1@S-1 and Pt1Cu3@S-1, corresponding to Pt:Cu precursor ratios of 3:1 and 1:3, respectively) and Pt3Cu1@MFI catalysts with Si/Al molar ratios of 274, 181, and 127 were synthesized. Chemical compositions of mother gels and resultant catalysts are summarized in Tables S2 and S3. All of these catalysts exhibited XRD patterns consistent with the MFI zeolite structure (Figure S9) and showed negligible XPS signatures of Pt and Cu (Figure S10), confirming the successful encapsulation of Pt–Cu nanoalloys in each case. HAADF-STEM directly observed the encapsulated nanoparticles with average diameters in the 1.2–1.4 nm range (Figures S11–S16), similar to the 1.2 nm size estimated for Pt1Cu1@S-1. CO-FTIR spectroscopy further verified that these variants all form Pt–Cu alloy sites (Figure 3G,H). In PtCu@S-1, as the Cu/(Pt + Cu) ratio increased, the ν_CO_ band corresponding to CO on Pt sites (band I after Ar purge) shifted to lower energy, indicating that Pt sites become increasingly electron-rich with higher Cu content, consistent with enhanced alloying effects. Meanwhile, the transient Cu–CO band (band II) observed during the purge exhibited a slight blueshift with a higher Cu content (Figure S17A). A correlation between the positions of bands I and II was observed across the different alloy compositions (Figure S17B), suggesting that the geometric and electronic structures of the alloy sites vary systematically with composition. On the other hand, introducing aluminum into the zeolite framework (decreasing Si/Al molar ratio) led to a blueshift of the Pt–CO band I, implying that the presence of framework Al (and the associated Brønsted acid sites) makes the Pt sites more electrophilic due to the confinement effects of micropores containing positively charged acid sites.

Al incorporation into the zeolite framework influences not only the electronic state of the encapsulated Pt–Cu nanoparticles but also the polarity of the surrounding pore space. To assess how framework Al concentration modulates the hydrophobicity of the zeolite cages that host the Pt–Cu alloy nanoparticles, we carried out continuous-flow CH_3_OH breakthrough experiments: a dilute CH_3_OH/Ar stream was passed through each catalyst bed at ambient temperature, and the effluent CH_3_OH concentration (*C*) relative to the inlet concentrations (*C*_0_) was recorded over time, yielding the breakthrough profiles (Figure 4A). The time required for the effluent to reach *C*/*C*_0_ = 0.05 was defined as the breakthrough time; these values were estimated for all catalysts and are compared in Figure 4B. A purely siliceous S-1 framework encapsulating Pt–Cu nanoparticles led to the earliest breakthrough, whereas breakthrough for the Pt3Cu1@MFI catalysts occurred progressively later as the Si/Al ratio decreases from 274 to 184 and 127. Quantitatively, the breakthrough time increased from 223–258 s for the siliceous catalysts to 340, 364, and 421 s for MFI with Si/Al = 274, 184, and 127, respectively. Because the three PtCu@S-1 catalysts differ only in alloy stoichiometry yet display nearly identical breakthrough times, host composition rather than the metal ratio governs CH_3_OH affinity, confirming that hydrophobicity is dictated by the concentration of framework Al. In short, the hydrophobicity of the reaction environment can be tuned by adjusting the framework Al content without compromising the encapsulation of the Pt–Cu alloy nanoparticles.

**Figure 4:**
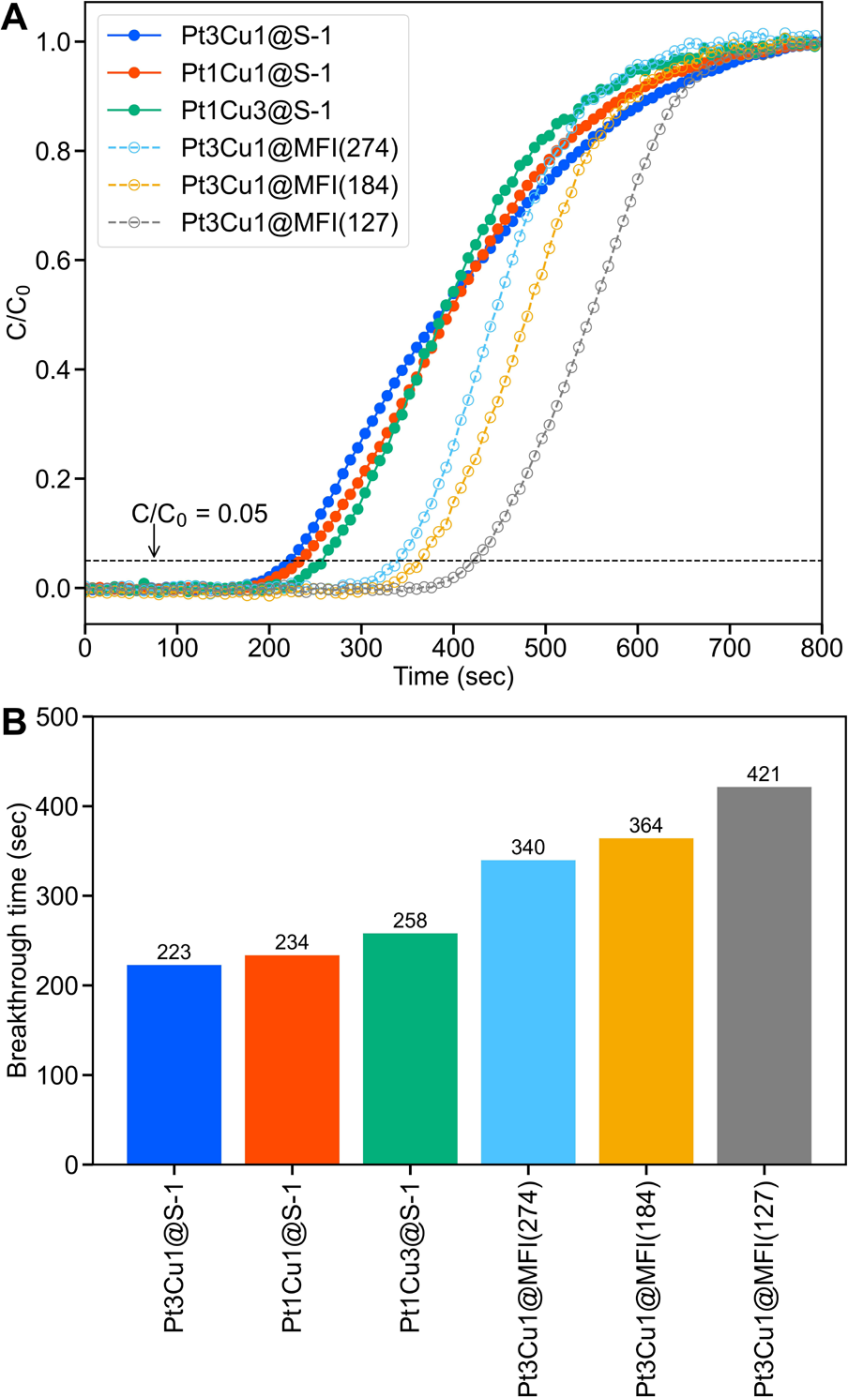
(A) CH_3_OH breakthrough curves for PtCu@S-1 with different Cu/Pt molar ratios and Pt3Cu1@MFI with different Si/Al molar ratios. The horizontal dashed line marks the breakthrough threshold (*C*/*C*_0_ = 0.05). (B) Comparison of the breakthrough times.

Accordingly, continuous modulation was achieved in both the structure and the electronic state of the Pt–Cu alloy species and their surrounding environment. The library of well-defined catalysts provides a solid foundation for investigating how composition and environment affect catalytic performance in CO-assisted oxidation of CH_4_.

### Catalytic Performance and Structure–Function Relationship

The catalytic performance of the aforementioned catalysts for the CO-assisted oxidation of CH_4_ to CH_3_OH was investigated. Activity assays were carried out in an aqueous medium at 150 °C for 1 h, using 20 bar CH_4_, 5 bar CO, and 3 bar O_2_ as reactant gases. Following the reaction, the liquid products were analyzed by ^1^H NMR spectroscopy. Representative spectra are available in Figure S18. It was found that C1 and C2 oxygenates were formed and their productivities and selectivities varied significantly with the catalyst employed.

Figure 5A compares the catalytic performance of PtCu@S-1 catalysts with varying alloy compositions as well as the monometallic Pt@S-1 and Cu@S-1. As the Cu content in the alloy increased (moving from Pt@S-1 to Pt3Cu1@S-1 to Pt1Cu1@S-1), the CH_3_OH productivity increased dramatically from 0.10 mmol/g_cat_/h (Pt@S-1) to 1.30 mmol/g_cat_/h (Pt3Cu1@S-1) and further to 1.44 mmol/g_cat_/h (Pt1Cu1@S-1). Along with this ∼14-fold increase in activity, the CH_3_OH selectivity improved from 31% (with Pt alone) to 94 and 95% for the 3:1 and 1:1 Pt–Cu alloys, respectively. However, an excess of Cu was detrimental: the Pt1Cu3@S-1 catalyst (with a 1:3 ratio) gave a lower CH_3_OH productivity (0.5 mmol/g_cat_/h) and a slightly reduced selectivity (87% CH_3_OH). Meanwhile, Cu@S-1 (which contains no Pt) showed no measurable activity for CH_4_ oxidation under these conditions. These results clearly demonstrate that both Pt and Cu are required in roughly equal proportions to achieve high activity and selectivity, a strong indication of synergy between Pt and Cu in the alloy. This is also true when the productivity is normalized by moles of Pt, further supporting the optimal composition (Table S5). The synergy observed here aligns with our design hypothesis that a Pt–Cu alloy can outperform the individual metals: neither Pt nor Cu alone was effective, but together, they achieved a remarkable outcome.

**Figure 5:**
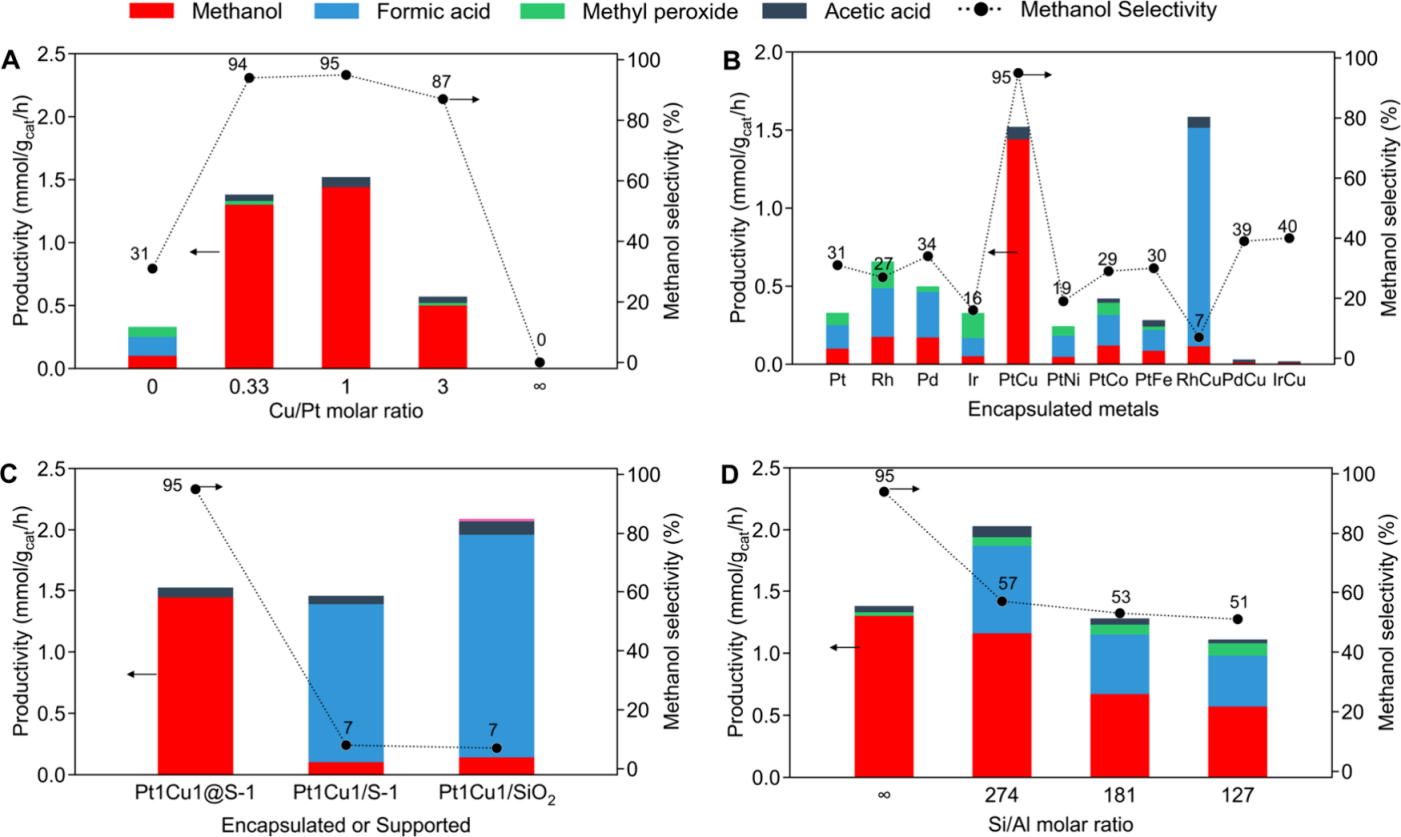
Structure–function relationship in the CO-assisted oxidation of CH_4_ over Pt–Cu nanoalloy-encapsulated zeolites. (A) Effect of the Cu/Pt molar ratio on oxygenate productivity and CH_3_OH selectivity. (B) Influence of noble metal and alloy composition within S-1. For bimetallic systems, a molar ratio of 1:1 was employed as Pt1Cu1@S-1. (C) Comparison of encapsulated and supported catalysts. (D) Effect of the Si/Al molar ratio. Reaction conditions: catalyst loading, 5 mg; solvent, 15 mL of H_2_O; reaction gas, 20 bar CH_4_ + 5 bar CO + 3 bar O_2_; temperature, 150 °C; reaction time, 1 h.

To verify that the Pt–Cu combination is indeed unique in promoting selective CH_4_ oxidation, a series of analogous bimetallic and monometallic catalysts using other metals was additionally synthesized and tested. These one-step hydrothermally prepared catalysts included Rh@S-1, RhCu@S-1, Pd@S-1, PdCu@S-1, Ir@S-1, IrCu@S-1, and PtFe@S-1, PtCo@S-1, and PtNi@S-1. The performance of these catalysts is summarized in Figure 5B. Only the Pt–Cu alloys (PtCu@S-1) exhibited high CH_3_OH productivity and selectivity. Catalysts containing only a single noble metal (Pt, Rh, Pd, or Ir) showed modest productivity (0.2–0.7 mmol/g_cat_/h) with poor selectivities (16–31% CH_3_OH), primarily yielding overoxidation products, HCOOH. The various bimetallic combinations (PtFe, PtCo, RhCu, PdCu, and IrCu) did show some degree of activity, but their methanol selectivities were only 7–40%. In fact, many of these bimetallics preferentially produced HCOOH, indicating that they did not solve the overoxidation issue. Only the Pt–Cu alloy encapsulated in S-1 achieved both high CH_3_OH productivity and high selectivity. Notably, for one of the least effective catalysts (RhCu@S-1), which exhibited activity comparable to that of PtCu@S-1 but predominantly yielded HCOOH, STEM and XPS analyses confirmed the successful encapsulation of Rh–Cu alloy nanoparticles (Figures S19–S21). This means the inferior performance of Rh–Cu alloy nanoparticles was not due to a failure in synthesis or lack of alloy formation, but rather an inherent difference in how Rh–Cu vs Pt–Cu function. These screening results demonstrate that the Pt–Cu pairing is uniquely effective for this reaction, validating our focus.

Next, the encapsulated Pt–Cu nanoalloy catalyst was compared with analogous catalysts in which Pt–Cu nanoalloys are supported on the external surface of S-1, to assess the significance of the zeolite confinement effect. Additionally, a high-surface SiO_2_-supported Pt–Cu catalyst with an average particle size of 1.5 nm was also prepared and used as the reference (Figures S22 and S23). Figure 5C compares the performance of Pt1Cu1@S-1 with Pt1Cu1/S-1 and Pt1Cu1/SiO_2_. The encapsulated Pt1Cu1@S-1 catalyst exhibited a CH_3_OH productivity of 1.44 mmol/g_cat_/h with 95% CH_3_OH selectivity, whereas both supported catalysts displayed a CH_3_OH productivity of less than 1/10th of this value (approximately 0.14 mmol/g_cat_/h) and a CH_3_OH selectivity of only 7%. The main product of the supported catalyst was HCOOH. This indicates that once CH_3_OH was formed, most of it was overoxidized to HCOOH. These results highlight that encapsulation within the zeolite micropores is critical for achieving high CH_3_OH selectivity. The hydrophobic micropores of S-1 facilitate the rapid expulsion of CH_3_OH, synthesized by the encapsulated Pt–Cu alloy nanoparticles, into the aqueous solvent, preventing further oxidation to HCOOH. In contrast, on open surfaces such as external S-1 and SiO_2_, CH_3_OH remains in prolonged contact with active sites, leading to continuous oxidation to HCOOH. What must not be overlooked here is that the overall formation rate of C1 oxygenates varies little among the three catalysts. The average nanoparticle diameters are 1.2 ± 0.4 nm for Pt1Cu1@S-1 and 1.5 ± 0.5 nm for Pt1Cu1/SiO_2_, whereas Pt1Cu1/S-1 contains significantly larger particles with a diameter of 3.0 ± 1.3 nm, implying substantial differences in the number of exposed metal sites. Yet, all three catalysts displayed almost identical total C1 oxygenate formation rates. This finding suggests that the particle size is not the decisive factor governing catalytic activity.

The effect of Brønsted acid sites in the zeolite (introduced by framework Al) on the catalytic performance was also investigated. Figure 5D compares PtCu@MFI catalysts with varying Si:Al molar ratios. When using purely siliceous S-1 (Si/Al = ∞, no acid sites), the catalyst gave 1.44 mmol/g_cat_/h CH_3_OH with 95% selectivity. Productivity increases when the ratio of Si/Al is 274, but CH_3_OH selectivity decreases due to the further oxidation of CH_3_OH. As the Si/Al molar ratio further decreases, productivity decreases, and excessive oxidation reactions also reduce the selectivity of CH_3_OH. These findings indicate that Brønsted acid sites (associated with Al in the framework) negatively impact selectivity, likely by promoting the further oxidation of CH_3_OH to HCOOH via acid-catalyzed pathways; acid-free support is essential for maximizing CH_3_OH selectivity, consistent with previous studies showing that the removal of acid sites or proton in aqueous solvent improves CH_3_OH selectivity.^14,34^ Furthermore, incorporating framework Al renders the zeolite channels more hydrophilic, hampering the prompt desorption of CH_3_OH from the reaction environment (Figure 4). The resulting longer residence time favors sequential CH_3_OH oxidation and therefore diminishes the CH_3_OH selectivity.

Figure 6 summarizes the CH_3_OH productivity (mol/mol_NM_/h) and CH_3_OH selectivity (%) of the best catalyst developed in this study (Pt1Cu1@S-1) with those of previously reported catalysts.^14–16,19,20,26,31,32^ In the literature systems, it has been challenging to enhance activity while maintaining a high selectivity (>90%). The maximum CH_3_OH productivity is limited to approximately 73 mol/mol_NM_/h reported for Au/MOR (0.07 wt % Au loading).^16^ In contrast, the Pt1Cu1@S-1 catalyst achieved a CH_3_OH productivity of 134 mol_methanol_/mol_NM_/h with 95% CH_3_OH selectivity, breaking the activity–selectivity limit. This remarkable catalytic performance underscores the importance of integrating the distinct catalytic functions of Pt and Cu within a confined, acid-free, hydrophobic environment that rapidly removes CH_3_OH and prevents overoxidation.

**Figure 6:**
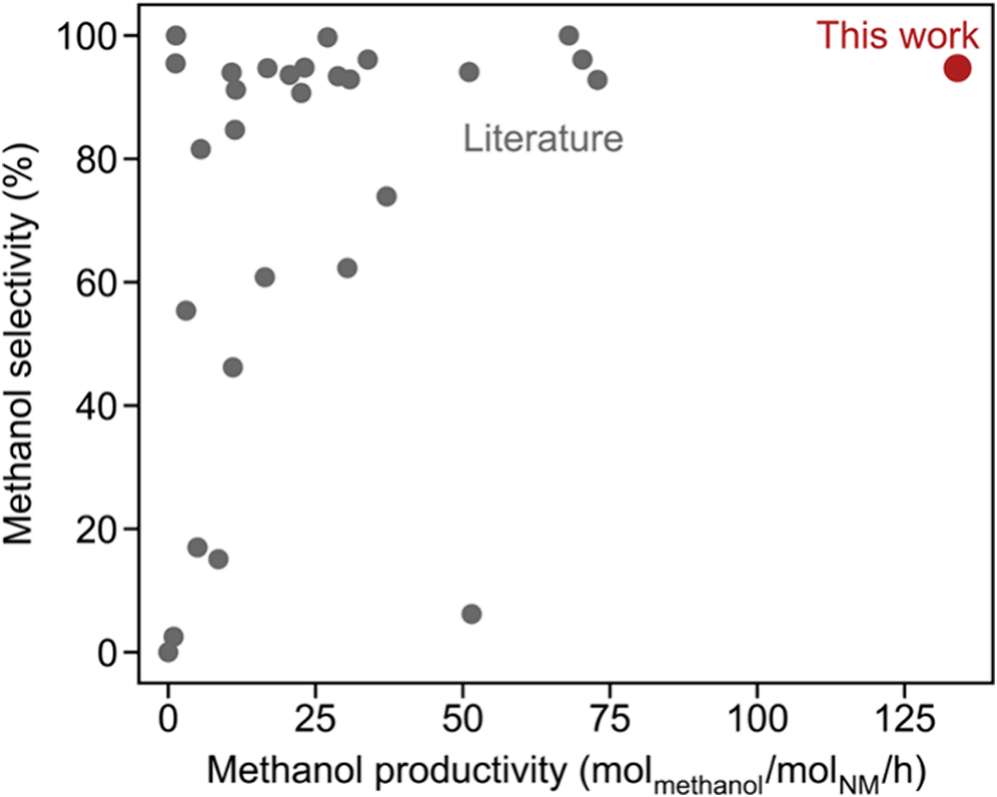
Comparison of Pt1Cu1@S-1 with literature catalysts in terms of CH_3_OH productivity and selectivity.

Catalyst recyclability was assessed over two consecutive batch runs (Figure S24). After reuse, the catalyst exhibited a 1.1–1.6-fold increase in the C1–C2 oxygenase formation rate but a moderate decline in CH_3_OH selectivity: 95% in the first run versus 78% for the second cycle (with no pretreatment) and 52% for the second (H_2_) cycle (prereduced with H_2_ at 500 °C). In CO-assisted oxidation of light alkanes, performance changes are frequently linked to nanoparticle sintering; we previously observed rapid Pt aggregation on r-TiO_2_ under analogous conditions, resulting in activity loss.^18^ Consistent with this precedent, HAADF–STEM reveals a modest particle growth from 1.2 ± 0.4 to 1.5 ± 0.5 nm (Figures S25 and S26), while XPS detects no Pt or Cu on the external zeolite surface (Figure S27), confirming that incipient sintering occurs within the micropores of S-1. Complementary CO-FTIR spectroscopy further revealed the irreversible surface structure changes associated with aggregation (Figure S28). Slight differences were detected in the XANES spectra of the catalysts before and after the reaction (Figure S29A,B), supporting the irreversible subtle changes in surface structure shown by CO-FTIR. On the other hand, *k* ^3^-weighted FT-EXAFS showed that the Pt–Cu and Pt–Pt backscattering peaks were retained after the reaction, which were comparable to those of the fresh catalysts (Figure S29C,D), suggesting that no extensive structural changes, such as dealloying, occurred during the reaction. Thus, the moderate decrease in CH_3_OH selectivity is more plausibly attributed to fine-scale surface modifications than to an overall change in the bulk structure of the alloy. Although further improvements in long-term durability, especially enhanced resistance to aggregation, are still necessary, the extremely high initial activity and selectivity of the Pt–Cu nanoparticles confined within the hydrophobic S-1 micropores validate our design strategy and provide a compelling platform for deeper mechanistic exploration.

### Mechanistic Insights

Having established the superior performance of the PtCu@S-1 catalyst, the reaction mechanism was subsequently investigated to elucidate how the Pt–Cu synergy operates. Focusing on the Pt1Cu1@S-1 catalyst, a series of kinetic experiments was conducted by varying the reactant partial pressures and reaction temperatures while simultaneously investigating potential reaction intermediates.

Figure 7 illustrates how the oxygenates productivity (mmol/g_cat_/h) and CH_3_OH selectivity (%) vary with CO or O_2_ pressures. Starting from the standard conditions (20 bar CH_4_ + 5 bar CO + 3 bar O_2_ at 150 °C), the CO partial pressure was varied between 0 and 7 bar while keeping CH_4_ at 20 bar and O_2_ at 3 bar (Figure 7A). Separately, the O_2_ partial pressure was varied between 0 and 6 bar with CH_4_ at 20 bar and CO at 5 bar (Figure 7B). When either CO or O_2_ was omitted, CH_3_OH was not formed, confirming that both coreactants are essential for the reaction. This observation is consistent with the CO-assisted oxidation mechanism proposed in earlier studies.^14–16^

**Figure 7:**
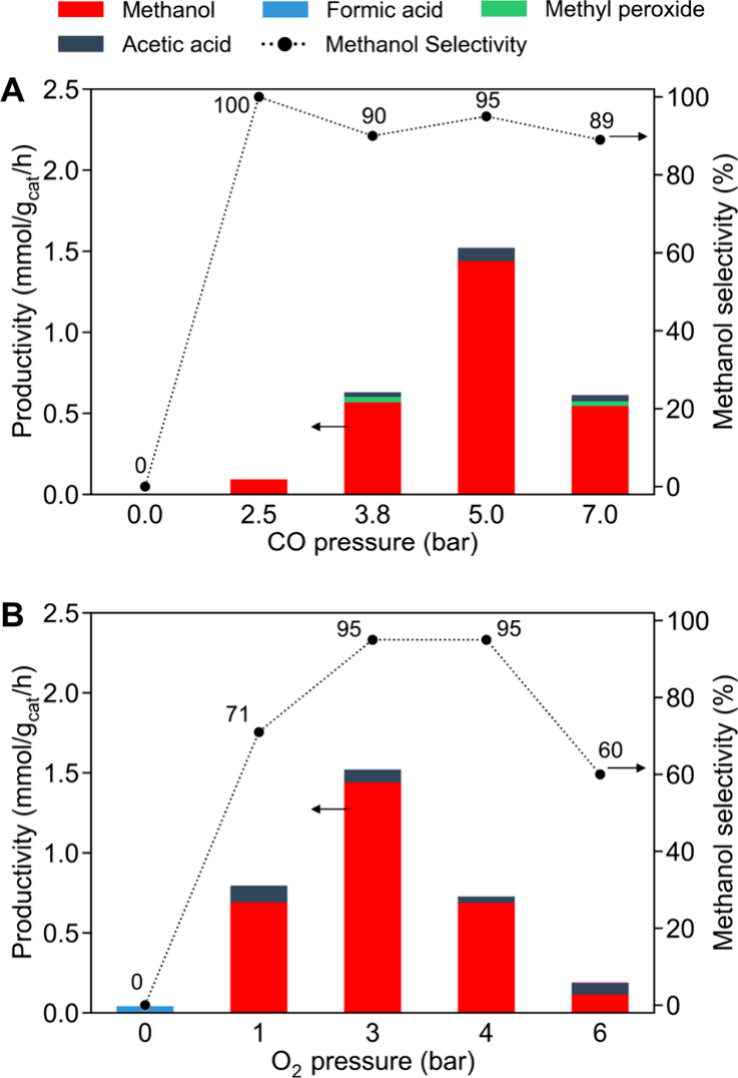
Effect of (A) CO and (B) O_2_ partial pressures on oxygenate productivity and CH_3_OH selectivity. Reaction conditions: catalyst loading, 5 mg; solvent, 15 mL of H_2_O; reaction gas, 20 bar CH_4_ + 0–7 bar CO + 3 bar O_2_ for (A) or 20 bar CH_4_ + 5 bar CO + 0–6 bar O_2_ for (B); temperature, 150 °C; reaction time, 1 h.

When CO was introduced, CH_3_OH production began once the CO pressure reached a threshold value. Specifically, as the CO pressure increased from 0 up to about 2.5 bar, CH_3_OH formation started with 100% selectivity. Further increasing the CO pressure from 2.5 to ∼5 bar led to a rapid rise in the CH_3_OH productivity, reaching a maximum of 1.44 mmol_CH3OH_/g_cat_/h. Interestingly, increasing the CO pressure beyond this optimum caused the overall oxygenate yield to decrease, suggesting that excessive CO inhibits the reaction. Excess CO may saturate active sites, hindering O_2_ activation and reducing oxidant availability. In other words, an optimal CO concentration is needed to effectively generate the active oxidizing species without poisoning the catalyst. It is known that CO helps maintain metal active sites in a low-valent (reduced) state, which is beneficial for sustained activity;^18,51^ however, too much CO could simply compete with CH_4_ or O_2_ binding. Therefore, an optimal CO concentration is vital. Importantly, CH_3_OH selectivity remained very high (>89%) over a broad range of CO pressures (2.5–7 bar). Even at the highest CO pressures, a significant shift toward CH_3_COOH formation was not observed, demonstrating robust selectivity for CH_3_OH.

In the case of O_2_, an optimal behavior was also observed. Starting from 0 bar O_2_, the CH_3_OH productivity increased as O_2_ pressure was raised to ∼3 bar, reaching a maximum value of oxygenate productivity of 1.44 mmol_methanol_/g_cat_/h with a 95% CH_3_OH selectivity. However, further increasing the O_2_ pressure to 4–6 bar decreased activity and lowered CH_3_OH selectivity to ∼60%. This indicates that an excess of O_2_ promotes the overoxidation of CH_3_OH to undesired products. Additionally, at high O_2_ partial pressure, the ability of CO to maintain the catalyst in a reduced state may be overwhelmed, leading to oxidative deactivation of active sites and a lower overall activity. These observations highlight that achieving high CH_3_OH yields requires careful balance in the supply of CO and O_2_.

To gain insight into the reaction pathway, the dependence of the reaction temperature on the product distribution was investigated. Figure 8A presents the selectivity for various products as a function of temperature (100–150 °C) over Pt1Cu1@S-1. At lower temperatures (e.g., 100–125 °C), a significant fraction of methyl hydroperoxide (CH_3_OOH) was observed in the postreaction solution, whereas at higher temperatures (≥135 °C), the CH_3_OOH signal diminished and more CH_3_OH was seen. Notably, the increase in CH_3_OOH selectivity at lower temperatures was correlated with a decrease in CH_3_OH selectivity. At 135–150 °C, a small amount (∼4 to 5%) of CH_3_COOH was detected, but no other liquid products were found. The temperature-dependent covariation of CH_3_OOH and CH_3_OH indicates that CH_3_OOH functions as an intermediate species in the pathway leading to CH_3_OH formation rather than as a terminal byproduct that does not further participate in the reaction sequence. In other words, at lower temperatures, CH_3_OOH accumulates because its conversion to CH_3_OH is slower, whereas at higher temperatures, CH_3_OOH is more rapidly converted to CH_3_OH, increasing the CH_3_OH selectivity. This mechanistic interpretation is consistent with our previous report on CO-assisted oxidation of ethane, where an ethyl hydroperoxide intermediate was implicated in the selective formation of ethanol.^18^ The implication for the present system is that CH_4_ is first oxidized to CH_3_OOH, which then decomposes or reacts further to yield CH_3_OH. To further substantiate this mechanistic hypothesis, a time-dependent selectivity profile was collected at 100 °C (Figure S30). After 3 h, the selectivity for CH_3_OOH decreased to 18%, while that for CH_3_OH increased to 82%. No other C1–C2 oxygenates were detected during this period, confirming the sequential conversion of CH_3_OOH to CH_3_OH under these conditions. When the reaction was extended to 6 h, the selectivities for CH_3_COOH and CH_3_CHO increased to 11 and 3%, respectively. This sequential conversion is expected to be attributed to the secondary reaction of the accumulated CH_3_OH with CO in the closed batch system.

**Figure 8:**
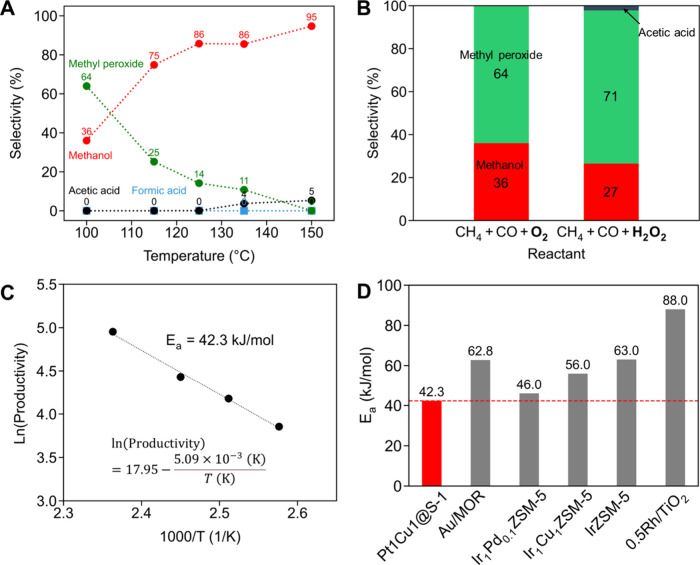
(A) Product selectivity at low temperatures. Reaction conditions: catalyst, 5 mg; solvent, 15 mL of H_2_O; reaction gas, 20 bar CH_4_ + 5 bar CO + 3 bar O_2_; reaction temperature, 100, 115, 125, 135, and 150 °C; reaction time, 1 h. (B) Comparison of product selectivities between the standard CH_4_ + CO+ O_2_ and CH_4_ + CO + H_2_O_2_ systems. Reaction conditions: catalyst, 5 mg; solvent, 15 mL of H_2_O or 16 mM H_2_O_2_ aq.; reaction gas, 20 bar CH_4_ + 5 bar CO + 3 bar O_2_ for the CH_4_ + CO + O_2_ system or 20 bar CH_4_ + 5 bar CO for the CH_4_ + CO + H_2_O_2_ system; temperature, 100 °C; reaction time, 1 h. (C) Arrhenius plot based on the productivity of total oxygenates. The dashed line represents the linear least-squares regression, and the corresponding fitting equation including the calculated activation energy is displayed within the figure. (D) Comparison of *E*_a_ between the present catalyst and previously reported catalysts.^15,16,32^

In the low-temperature regime, the productivity of total oxygenates produced a linear Arrhenius plot (Figure 8C). From the slope, the apparent activation energy (*E*_a_) was estimated as 42 kJ/mol. This value is the lowest among the literature data regarding the CO-assisted oxidation of CH_4_ (Figure 8D).^15,16,32^ The unusually low *E*_a_ in our system aligns with its exceptional activity (Figure 6) and provides further evidence of beneficial cooperation between Pt and Cu within hydrophobic micropores of S-1.

One key question is what oxidizing species are responsible for the initial conversion of CH_4_ to CH_3_OOH. A plausible hypothesis is that H_2_O_2_ is generated in situ from O_2_ and a source of H_2_ and that thus-formed H_2_O_2_ oxidizes CH_4_ to CH_3_OOH. To verify this hypothesis, the amount of H_2_O_2_ present in the aqueous solution after the reaction with CO and O_2_ was quantified using a Ce^4+^/Ce^3+^ titration method. Because H_2_O_2_ readily undergoes self-decomposition, the reaction temperature was set to a relatively low temperature (100 °C) to minimize its loss. The formation of H_2_O_2_ was evidenced by a distinct color change in the indicator (Figure S31), and its productivity was determined to be 4.6 × 10^–2^ mmol/g_cat_/h, which is on a similar order of magnitude as the oxygenate productivity observed in the CH_4_ + CO + O_2_ system at 100 °C (∼0.17 mmol/g_cat_/h). This relatively low H_2_O_2_ productivity may be attributed to its self-decomposition; it is expected that under CH_4_ reaction conditions, any H_2_O_2_ formed would be immediately consumed by CH_4_ oxidation, so the steady-state concentration of H_2_O_2_ remains low, thereby diminishing the impact of its decomposition.

To confirm that in situ generated H_2_O_2_ can indeed serve as the oxidant promoting CH_4_ oxidation, a controlled experiment was conducted in which O_2_ under standard CH_4_ + CO + O_2_ conditions was replaced by H_2_O_2_, keeping all other reaction parameters identical. Product selectivities were then compared between those of the standard CH_4_ + CO + O_2_ system and the CH_4_ + CO + H_2_O_2_ system. As shown in Figure 8B, both of the conditions gave similar selectivity, both producing primarily CH_3_OH and CH_3_OOH in comparable ratios. This result supports the above hypothesis that H_2_O_2_ produced in situ during a standard reaction (in the presence of CH_4_, CO, and O_2_) indeed acts as the oxidant in this selective oxidation process.

A key remaining question is the origin of hydrogen for H_2_O_2_ generation in this system. One plausible route is the water-gas shift (WGS) reaction: CO + H_2_O → CO_2_ + H_2_, which can occur under the present catalytic conditions in the presence of water as the solvent. We verified the formation of H_2_ gas from CO and H_2_O (solvent) over the Pt1Cu1@S-1 catalyst using gas chromatography (Figure S32), where the reaction was conducted at 150 °C, i.e., the same temperature employed to boost oxidation of CH_4_ to CH_3_OH. The measured H_2_ productivity was 1.0 mmol/g_cat_/h, which was almost comparable with that of total oxygenates (Table S5), thereby supporting the idea that the H_2_ generated via the WGS reaction supplies the hydrogen required for in situ H_2_O_2_ production.

Accordingly, the complete proposed reaction mechanism is illustrated in Figure 9. In this mechanism, CO reacts with water (the solvent) on the catalyst to generate H_2_, which then react with O_2_ to form H_2_O_2_. Finally, H_2_O_2_ oxidizes CH_4_ to yield CH_3_OOH, which subsequently decomposes into CH_3_OH. This mechanism aligns with the observation that both CO and O_2_ are required to catalyze the partial oxidation of CH_4_ to CH_3_OH (refer to Figure 7).

**Figure 9:**
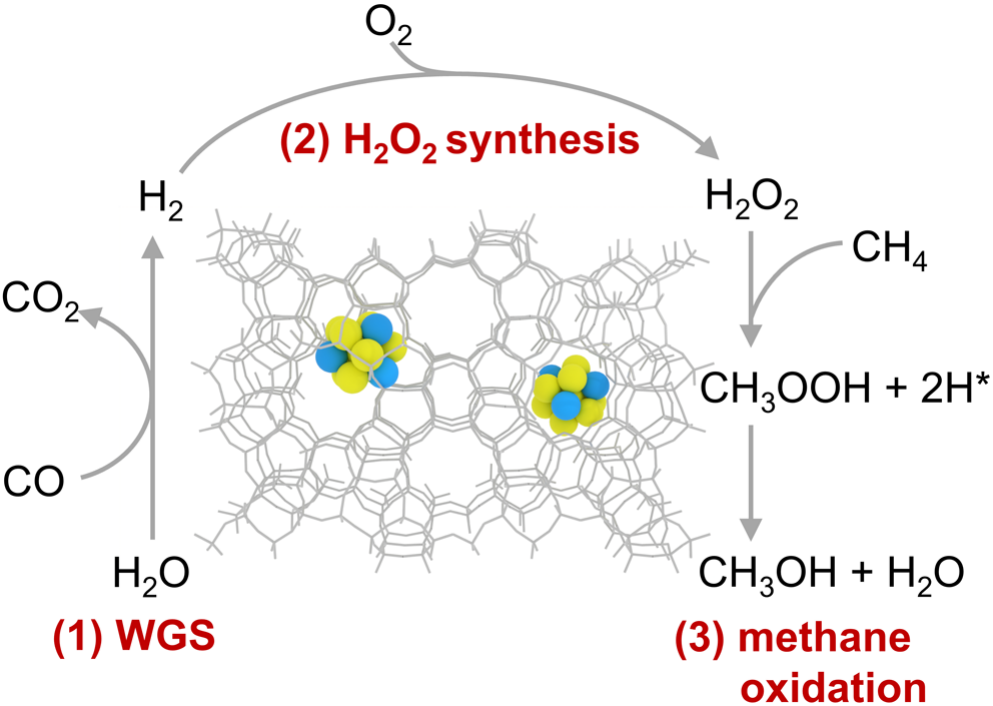
Proposed mechanism of the CO-assisted oxidation of CH_4_ to CH_3_OH catalyzed by PtCu@S-1.

An alternative mechanistic proposal in the literature is that OH^•^ generated from H_2_O_2_ or other sources are the actual species that convert CH_4_ to form CH_3_OH.^16^ To verify whether such a radical mechanism operates in our system, electron spin resonance (ESR) experiments were performed using a radical trap (5,5-dimethyl-1-pyrroline *N*-oxide, DMPO). If OH^•^ were formed during the reaction, then they would adduct with DMPO to form a DMPO–OH spin adduct, which can be detected as a characteristic quartet signal in the ESR spectrum. When the CH_4_ + CO + O_2_ reaction was conducted in the presence of aqueous DMPO, a weak 1:2:2:1 quartet signal derived from the DMPO–OH adduct was indeed detected, though its intensity was very low (Figure S33, red line). An OH^•^ productivity of only ∼0.4 × 10^–4^ mmol/g_cat_/h was revealed by fitting analysis, which is over three orders of magnitude lower than the oxygenate productivity (∼0.17 mmol/g_cat_/h) at the same temperature. It is indicated by this extremely low OH^•^ productivity that even if all detected OH^•^ were involved in the reaction, their contribution would be negligible. Furthermore, even when a large excess of the OH^•^ scavenger (30 mmol/g_cat_ of Na_2_SO_3_, which is ∼1000 times the detected amount of OH^•^) was added, no significant change in productivity and selectivity was observed (Figure S34). By contrast, a previously reported OH^•^-mediated pathway in the Au/MOR catalyst system was inhibited by 63% after the addition of only 4 mmol/g_cat_ of Na_2_SO_3_.^16^ Therefore, the negligible effect of the OH^•^ scavenger and the extremely low productivity of OH^•^, observed in the present study, suggest that an OH^•^-mediated mechanism does not govern CH_4_ oxidation over Pt1Cu1@S-1.

## Conclusions

We have demonstrated that encapsulating Pt–Cu nanoalloys within a hydrophobic, acid-free S-1 matrix enables low-temperature CH_4_ oxidation to CH_3_OH with a record productivity and nearly 100% selectivity. Comparative studies with model catalysts highlight the importance of precisely tuning alloy compositions and reaction environments of the encapsulated nanoalloys for optimizing activity and selectivity. The mechanistic insights into how hydrophobic, acid-free micropores promote CH_3_OH desorption and supprese overoxidation demonstrate the high potential of catalyst design that synergistically couple metal functionality with tailored pore architecture for efficient CH_3_OH production. Our findings suggest that further refinements of alloy composition and pore engineering can yield even greater control over product selectivity and productivity. In particular, establishing a synthesis protocol that embeds nanoparticles with the target alloy composition inside zeolite pores while simultaneously minimizing the density of Si–OH defects, which govern framework hydrophobicity, will be increasingly important. Because the present study did not completely eliminate these Si–OH groups (about 10% of the Q3 band was observed in ^29^Si MAS NMR, Figure S2), further improvements in hydrophobicity and therefore in catalytic performances can be anticipated by reducing the defect density. Furthermore, alloying the encapsulated nanoalloys with elements that improve their durability would address the stability limitations identified in this study. These strategies can be extended to other C–H bond activation processes. Furthermore, the results obtained here not only deepen our understanding of how CH_4_ can be directly converted to CH_3_OH but also inspire a broader exploration of hierarchical multifunctional catalysts for industrially relevant oxidation reactions. They are thus expected to open a promising avenue for more sustainable, economically viable routes to oxygenated fuels and chemicals, promoting continued advances in catalytic science to meet global energy and environmental challenges.

## Supplementary Material



## Notes

Experimental details, additional catalytic data, additional characterization data, and comparison with the literature catalysts (PDF)
