# Supplementary material for: Hydrophobic, Acid-Free Zeolite-Confined Pt–Cu Nanoalloys Break Activity–Selectivity Limits in Low-Temperature Methane-to-Methanol Oxidation
Source: J Am Chem Soc. 2025 Aug 11;147(33):30009–21. doi: 10.1021/jacs.5c07414 (PMC12371880; doi:10.1021/jacs.5c07414)
Supplement: Supplementary file 1 [file ja5c07414_si_001.pdf]

Supporting Information

Hydrophobic, Acid-Free Zeolite-Confined Pt–Cu Nanoalloys Break Activity–Selectivity Limits in Low-Temperature Methane-to-Methanol Oxidation

Akira Oda,*,†,‡ Koyo Ichino,† Yuta Yamamoto,§ Takeshi Ohtsu,† Wei Shi,† Yoshiharu Sawada,‖ Jun Kumagai,§ Kyoichi Sawabe,† Atsushi Satsuma†

†*Department of Materials Chemistry, Graduate School of Engineering, Nagoya University, Nagoya 464-8603, Japan.*

‡*Institute for Catalysis, Hokkaido University, Sapporo 001-0021, Japan.*

§*Institute of Materials and Systems for Sustainability, Nagoya University, Nagoya 464-8603, Japan.*

‖*Technical Center, Nagoya University, Nagoya, Aichi 464-8601, Japan.*

*Email: akira@cat.hokudai.ac.jp

Table of Contents

Table of Contents........ S2
Materials and Methods........ S3
Supporting Data........ S9
References........ S49

Materials and Methods

Materials. $Pt(NO_3)_2$ (Cataler Co., Ltd., 8.6 wt% in HNO_3 solution), $PtCl_2$ (FUJIFILM Wako Chemicals Co., Ltd., 98.0%), $Ir(NO_3)_3$ (Furuya Metal Co., Ltd, 100g/L), $RhCl_3 \cdot 3H_2O$ (FUJIFILM Wako Chemicals Co., Ltd.), $PdCl_2$ (FUJIFILM Wako Chemicals Co., Ltd., >99%), $CuCl_2$ (Kishida Chemical Co., Ltd.), $Cu(NO_3)_2 \cdot 3H_2O$ (Kishida Chemical Co., Ltd., 99.5%), $Ni(NO_3)_2 \cdot 3H_2O$ (Kishida Chemical Co., Ltd., 98.0%), $Co(NO_3)_2 \cdot 3H_2O$ (Kishida Chemical Co., Ltd., > 98.0%), tetraethyl orthosilicate (Tokyo Chemical Industry Co., Ltd., 98%), tetrapropylammonium hydroxide (Tokyo Chemical Industry Co., Ltd., 20–25%), ethylenediamine (Sakai Chemical Industry Co., Ltd., >99%), HF (FUJIFILM Wako Chemicals Co., Ltd., 46–48%), H_3BO_3 (FUJIFILM Wako Chemicals Co., Ltd., 99.5%), HCl (Kishida Chemical Co., Ltd., 35%), HNO_3 (Kishida Chemical Co., Ltd., 60%), ZSM-5 (NH_4^+-type, Si/Al = 20, HSZ-840NHA, TOSO Co., Ltd.,), SiO_2 (Cariact-G6, Fuji Silysia, 3 μm), CH_4 (Takachiho Chemical Industrial Co., Ltd., 99.99%), CO (SogoKariya sanso, >99.95%), O_2 (SogoKariya sanso, 99.99%), D_2O (FUJIFILM Wako Chemicals Co., Ltd.), DSS-d6 NMR standard (FUJIFILM Wako Chemicals Co., Ltd., 99.0%), $Na_2SO_3 \cdot 7H_2O$ (Kanto Chemical Co., Inc., >95.0%), CH_3OH (Kishida Chemical Co., Ltd., 99.8%).

Preparation of Ethylenediamine Complexes. $PtCl_2$ (240 mg) or $CuCl_2$ (121 mg) was dispersed in 18 mL of distilled water and stirred in a water bath at 80 °C for 10 min. Subsequently, 2 mL of ethylenediamine was added to obtain a final concentration of 0.045 M ethylenediamine complex solution. Similarly, ethylenediamine complexes of Fe, Co, Ni, Pd, Rh, and Ir were prepared. For Ir, Ni, and Co, ethylenediamine complexes could not be prepared using chloride precursors; thus, nitrate complexes were used as precursors.

One-Step Hydrothermal Synthesis of Metal-Encapsulated Zeolites. First, a 50 mL polypropylene bottle was charged with a magnetic stirrer, distilled water (13.5 g), and tetrapropylammonium hydroxide (14 g), followed by stirring for 10 min. A predetermined amount of metal precursor solution was then added. For the synthesis of MFI-type zeolite with framework Al sites, aluminum isopropoxide was additionally introduced. Subsequently, tetraethyl orthosilicate (8.32 g) was added, and the mixture was stirred at room temperature for 6 h to prepare the precursor gel. The composition of the precursor gel for each catalyst is listed in **Table S2**. The obtained precursor gel was transferred into a 100 mL inner vessel of a hydrothermal synthesis reactor, which was then sealed. The sealed reactor was placed in a preheated oven at 170 °C and maintained for 72 h to facilitate the hydrothermal reaction. After the reaction, the reactor was rapidly cooled with water, and the solid product was recovered via centrifugation at 6000 rpm for 5 min. The collected sample was redispersed in 60 mL of distilled water, and the centrifugation and washing process was repeated five times. After washing, the sample was dried overnight at 80 °C under ambient conditions. Following drying, the catalyst was subjected to reduction treatment under H_2 flow (20 mL/min) at 500 °C for 2 h (heating rate: 10 °C/min). This pretreatment was conducted immediately before activity assay and characterization

Preparation of PtCu-Supported S-1 Catalyst. Metal-free S-1 was synthesized following the same procedure as the one-step hydrothermal method for metal-encapsulated zeolites except for use of metal precursor and

ethylenediamine. The obtained S-1 was subsequently subjected to an incipient wetness impregnation method, where Pt and Cu nitrates were introduced at a 1:1 molar ratio. The total metal loading was adjusted to match the composition of Pt1Cu1@S-1 determined by ICP-OES (**Table S3**). For the impregnation, S-1 was placed in a separable flask, followed by the addition of distilled water and a predetermined amount of nitrate aqueous solutions of Pt and Cu. A magnetic stirrer was added into the solution, and the mixture was stirred at room temperature for 1 h. The solvent was then removed using a rotary evaporator at 80 °C, and the resultant solid residue was further dried overnight at 80 °C under ambient conditions. After drying, the catalyst was collected from the separable flask. Before activity assay and characterization, a reduction treatment was performed under H_2 flow (20 mL/min) at 500 °C for 2 h (heating rate: 10 °C/min). Note: in addition to this procedure, we also prepared the supported catalyst using ethylenediamine complexes of Pt and Cu as the metal precursors and examined whether the choice of precursor affected catalytic performance; however, no differences in either activity or selectivity were observed.

ICP-OES. The metal content in the catalysts was determined using an ICP-OES (SPS7800, Hitachi High-Tech Science). A 25 mg catalyst was dissolved in 4 mL of hydrofluoric acid, followed by the addition of 46 mL of saturated boric acid solution, adjusting the final volume to 50 mL. The resulting solution was introduced into the ICP-OES system to quantify the concentration of each metal element. For quantification, a calibration curve was made using metal nitrate standard solutions with concentrations of 0, 3, 5, and 10 ppm (mg/L). The obtained calibration curve exhibited a linearity, with a determination coefficient of $0.999 \leq R^2 \leq 1.000$. Based on the measured values, the metal content in each catalyst was calculated.

HAADF-STEM. The direct observation of the metal nanoparticles encapsulated within zeolite or supported on zeolite was conducted using HAADF-STEM with a JEM-ARM200F (JEOL) operated at an acceleration voltage of 200 kV. Prior to observation, the catalysts were subjected to H_2 reduction at 500 °C for 2 h under a flow of 20 mL/min. A small amount of the catalyst was collected using a spatula, transferred to a screw vial, and suspended in 3 mL of H_2O. The suspension was treated by ultrasonication for 30 min, and the supernatant was collected using a Pasteur pipette. Three droplets of the suspension were drop-cast onto a 200 mesh Cu collodion grid (EM Japan) and dried at ambient pressure for 30 min. Subsequently, the grids were placed in a grid case, followed by vacuum drying overnight in a desiccator. Finally, the grids were mounted on a single-axis tilt holder for imaging. Micrographs were processed with a Gatan Microscopy Suite software.

CO-FTIR. To evaluate the geometric and electronic states of the catalyst surface sites, CO-FTIR spectroscopy was conducted using a JASCO FT/IR-6600 spectrometer. Measurements were performed in transmission mode, with a measurement range of 4000–1250 cm^{-1}, a resolution of 2 cm^{-1}, and 64 accumulations (16 accumulations for time-resolved measurements). A high-intensity ceramic light source (7800–50 cm^{-1}) and an MCT detector were used. The catalyst was pressed into a 10 mm diameter pellet and placed in a quartz cell for measurement. Prior to the measurement, the catalyst was reduced under 100% H_2 flow (20 mL/min) at 500 °C for 2 h (heating rate: 10 °C/min).

After reduction, the catalyst was cooled from 500 °C to 50 °C under Ar flow (90 mL/min) and held at 50 °C for 10 min. The background spectrum was then collected. For CO adsorption, 0.4% CO/Ar (100 mL/min) was introduced for 30 min. After that, the CO gas flow was stopped; only Ar (90 mL/min) was flowed for 30 min to observe the desorption behavior of the adsorbed CO species. To track dynamic changes, time-resolved measurements (121 scans) were performed for the desorption process. The measurement interval was 16 sec.

XAFS. XAFS measurements were performed to investigate the local structure and electronic state of the Pt and Cu located within/on the S-1. Prior to the measurements, 50 mg of catalyst was pressed into a 10 mm diameter pellet and subjected to reduction treatment at 500 °C (heating rate: 10 °C/min) for 2 h under a 20 mL/min H_2 flow. The reduced pellets were subsequently sealed in a film within a glove box to prevent air exposure. Pt L_{III}- and Cu K-edge XAFS measurements were conducted at the Aichi Synchrotron Radiation Center (BL5S1 beamline) using a Si(111) monochromator. During each measurement, Pt/Cu foil was simultaneously measured as a reference for energy calibration. The acquired spectra were energy-calibrated, normalized, and Fourier-transformed with Athena software.[1] WT analysis was performed using the free software provided by ESRF.[2,3] The Morlet wavelet ($\sigma = 1$, $\kappa = 15$) was employed as the mother wavelet. For both FT and WT, the $k^3\chi(k)$ function in the range of $3.0 < k < 13.0$ Å^{-1} was used.

XPS. XPS was performed to confirm whether metals were encapsulated within the zeolite pores. Measurements were conducted using a PHI Quantes (ULVAC-PHI) spectrometer with Al Kα radiation as the X-ray source. The analysis targeted Pt 4f, Cu 2p, Rh 3d, and C 1s signals in the catalyst after pretreatment. For pretreatment, the catalysts were subjected to H_2 reduction under a 20 mL/min flow at 500 °C for 2 h (heating rate: 10 °C/min). The catalysts were then uniformly dispersed onto an indium foil substrate, and excess material was removed using a blower before placement on the sample stage. The sample height was adjusted to maximize photoelectron emission. XPS spectra were acquired with a pass energy of 224 eV, an energy step size of 0.05 eV, and a dwell time of 20 ms/step. The obtained data were analyzed using MultiPak (ULVAC-PHI), and energy calibration was performed using the binding energy of C 1s signal as a reference.

XRD. XRD analysis was performed to investigate the crystalline structure of the prepared catalysts. The measurements were conducted using a MiniFlex600-c (Rigaku) diffractometer with Cu Kα radiation as the X-ray source. The analysis was carried out at room temperature using catalysts pre-reduced with H_2. For reduction pretreatment, the catalysts were heated to 500 °C under 100% H_2 flow (20 mL/min) at a ramp rate of 10 °C/min, held at this temperature for 2 h, and then cooled to room temperature. XRD measurements were performed under the following conditions: an accelerating voltage of 40 kV, a tube current of 15 mA, a scan range of 3–90°, a scan step of 0.01°, and a step speed of 10°/min.

Simultaneous Thermogravimetry and Differential Thermal Analysis (TG-DTA). TG-DTA was performed on either calcined Pt1Cu1@S-1 or the uncalcined precursor (S-1 containing ethylenediamine,

ethylenediamine complexes of Pt and Cu, and tetrapropylammonium cations). 10 mg of the sample was weighed into an aluminum crucible and loaded into the analyzer (Thermo Plus EVO2 TG–DTA 8122, Rigaku). The sample was heated at a rate of 10 °C min^{-1} under a flowing 20 % O_2/Ar atmosphere while weight loss, temperature, and heat flow were monitored.

Nitrogen Adsorption-Desorption Isotherms. Nitrogen adsorption–desorption isotherms were recorded at −196 °C (77 K) on a BELSORP-mini II (MicrotracBEL). Before analysis, ca. 200 mg of zeolite powder was outgassed under high vacuum (< 3×10^{-3} Pa) at 350 °C for 2 h to eliminate physisorbed moisture. Specific surface areas were evaluated from the adsorption isotherms by applying the Brunauer–Emmett–Teller (BET) model over the relative pressure window P/P_0 = 0.05–0.30. Micropore volume was estimated using t-plot analysis.

^{29}Si MAS NMR Spectroscopy. Solid-state ^{29}Si MAS NMR spectra were recorded using a spectrometer (AVANCE NEO 500, Bruker) operating at a magnetic field of 11.7T with an operational frequency for ^{29}Si of 99.3 MHz. Powdered samples were packed into 4 mm-diameter zirconia rotors and spun at a rate of 14 kHz at the magic angle. Spectra were acquired with 3.3 μs excitation pulses, a 60 s recycle delay, and 1000 scans. Chemical shifts were externally referenced to octa(dimethylsiloxy)-octasilsesquioxane (δ = −12.6 ppm).

CH_3OH Breakthrough Curve. Breakthrough experiments with CH_3OH were conducted on an FTIR spectrometer (IRSpirit, Shimadzu) equipped with a triglycine sulfate detector and a gas-phase transmission cell (optical path length = 10 cm, KRS-5 windows). Approximately 50 mg of catalyst (particle size 300–600 μm) was loaded into a U-shaped quartz tube and prereduced in flowing H_2 (20 mL/min). The temperature was ramped from room temperature to 500 °C at 10 °C/min and held for 2 h. After cooling the catalyst bed to 50 °C, the carrier gas was switched to Ar. A feed consisting of 4 vol % CH_3OH in Ar (total flow 13 mL/min) was generated by passing Ar through a CH_3OH solution cooled at 0 °C and introduced into the system. The effluent was diluted downstream with 87 mL/min of Ar and continuously analyzed by FTIR spectroscopy. Spectra were recorded in the 4000–400 cm^{-1} region at a resolution of 4 cm^{-1}, collecting one scan per spectrum with an interval of 8 sec. The breakthrough profile was obtained by monitoring the absorbance of the C–O stretching band of CH_3OH at 1032.4 cm^{-1} as a function of time on stream.

Activity Assay. Prior to the reaction, 5 mg of catalyst was subjected to H_2 reduction at 500 °C for 2 h in a test tube. After reduction, 15 mL of H_2O and a magnetic stirrer were added to the test tube, followed by ultrasonication for 2 min to achieve uniform dispersion of the catalyst. The prepared catalyst suspension was transferred to a reactor, which was subsequently filled with CO, O_2, and CH_4. Typically, the partial pressures of CO, O_2, and CH_4 were set to 5 bar, 3 bar, and 20 bar, respectively. The reactor was then placed in an oil bath at 150 °C and stirred at 1500 rpm for 1 h to facilitate the reaction. After the reaction, the supernatant was filtered, and 0.7 μL of the filtrate was mixed with 0.1 μL of a 0.02 wt% DSS standard solution (D_2O solvent) in an NMR tube. Liquid-phase products were analyzed using ^{1}H NMR spectroscopy (AVANCE IIIHD 500 MHz, Bruker), and their concentrations were quantified based on

the DSS internal standard. The chemical shifts of C**H**$_3$OH (singlet), **H**COOH (singlet), C**H**$_3$CHO (doublet), C**H**$_3$COOH (singlet), and C**H**$_3$OOH (singlet) were used for quantification. No other liquid-phase oxygenates were detected under the present reaction conditions.

The productivity and selectivity were defined according to the following equations:

$$\text{Productivity (mmol/g}_{\text{cat}}\text{/h)} = \frac{\text{moles of C1 and C2 oxygenates (mmol)}}{\text{weight of catalyst (g}_{\text{cat}}) \times \text{reaction time (h)}}$$

$$S_{CH_3OH}(\%) = \frac{\text{moles of } CH_3OH \text{ (mol)}}{\text{total moles of liquid- phase products (mol)}} \times 100$$

$$S_{CH_3OOH}(\%) = \frac{\text{moles of } CH_3OOH \text{ (mol)}}{\text{total moles of liquid- phase products (mol)}} \times 100$$

$$S_{HCOOH}(\%) = \frac{\text{moles of HCOOH (mol)}}{\text{total moles of liquid- phase products (mol)}} \times 100$$

$$S_{CH_3COOH}(\%) = \frac{\text{moles of } CH_3COOH \text{ (mol)}}{\text{total moles of liquid- phase products (mol)}} \times 100$$

To compare with the literature catalyst, methanol productivity based on moles of noble metal was also estimated by the following equation:

$$\text{Methanol productivity (mol}_{\text{methanol}}\text{/mol}_{\text{NM}}\text{/h)} = \frac{\text{moles of methanol (mmol)}}{\text{moles of noble metal (mol}_{\text{NM}}) \times \text{reaction time (h)}}$$

, where moles of noble metal was determined by ICP-OES. As discussed in the manuscript, PtCu@S-1 and Pt@S-1 were both effective for the selective oxidation of methane, whereas Cu@S-1 showed no activity at all. This clearly indicates that Pt is the indispensable catalytic element for the reaction. Therefore, we normalized methanol formation rates by the mole of true active element (Pt) and compared with the literature catalysts.

CO_2 is generated from both the oxidation of added CO and the overoxidation of CH_4. Since the produced CO_2 dissolves in the aqueous solvent, its precise quantification is challenging. Therefore, in this study, selectivity was calculated based solely on the liquid-phase oxygenates, without considering CO_2 formation.

The concentration of H_2O_2 in the post-reaction solution was determined via titration with a 0.1 M $Ce(SO_4)_2$ aqueous solution. First, a 0.5 M $Ce(SO_4)_2$ solution in 1 M H_2SO_4 was prepared by mixing equal volumes of 1 M $Ce(SO_4)_2$ solution and 2 M H_2SO_4. For the quantification of H_2O_2 in the post-reaction solution, 3 mL of the solution was extracted into a screw-tube bottle, and a drop of ferroin indicator was added, turning the solution red. A 0.1 M $Ce(SO_4)_2$ solution was then titrated dropwise (10 μL increments) until the solution changed from red to pale blue. The H_2O_2 concentration was determined based on the total volume of titrant added and the calibration curve.

The H_2 generated via the WGS reaction was quantified using gas chromatography with barrier ionization detector and a RT-Msieve 5A capillary column. The gas samples were collected from the reactor using a 100 μL syringe after the reaction.

ESR. ESR spectroscopy was employed to investigate radical species formed during the catalytic reaction. The analysis focused on detecting •OH, •CH_3, and •OOH radicals in the post-reaction solution. For catalyst pretreatment, the catalysts were subjected to H_2 reduction under a 20 mL/min flow at 500 °C for 2 h (heating rate: 10 °C/min). The reduced catalyst was then dispersed in 7.5 mL of distilled water and 7.5 mL of 20 mM DMPO solution, followed by the addition of a magnetic stirrer. The mixture was ultrasonicated for 2 min before being transferred to a pressure-resistant vessel. The air within the reactor was replaced with O_2 by purging three times at 3.0 bar, and then the high-pressure mixture-gas was prepared by subsequent purging CO (5.0 bar), and CH_4 (20 bar) inside the vessel. The reaction was conducted at 100 °C with stirring at 1500 rpm for 1 h in an oil bath. After the reaction, the solution was filtered, and 1 mL of the filtrate was introduced into an ESR cell for liquid-phase analysis. Spectra were recorded at room temperature on a JES-RE1X spectrometer (JEOL) under the following settings: power = 1 mW, center field = 335.8 mT, field width = ±5 mT, sweep time = 8 min, frequency = 9.3965 GHz, modulation frequency = 100 kHz, modulation width = 0.1 mT, and time constant = 0.3 s. Four scans were averaged, and a Mn standard was measured simultaneously for calibration. Radical concentrations were quantified from the integrated spin-adduct signal. Data analysis was performed using a EasySpin software.

Supporting Data

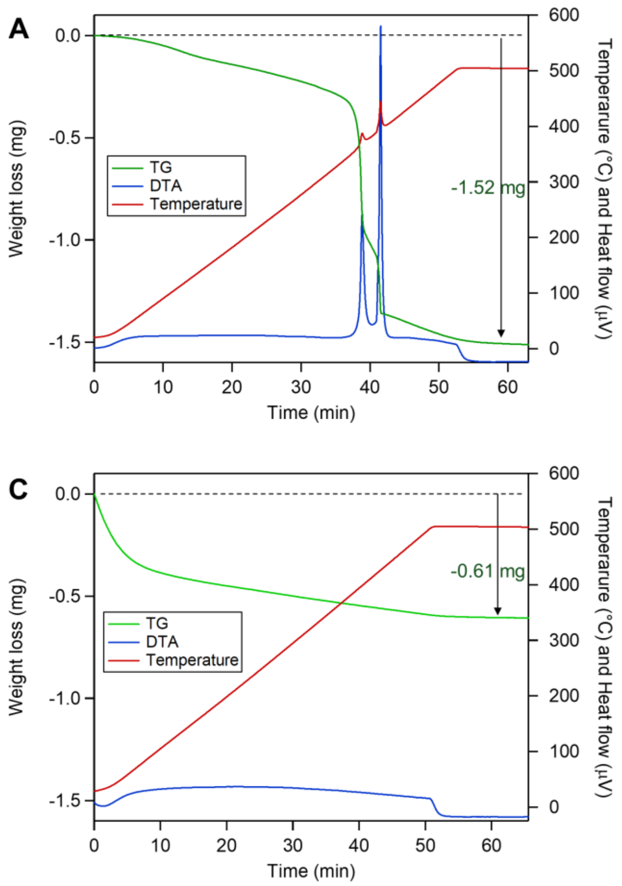

Figure S1. TG-DTA curves for Pt1Cu1@S-1 (A) before and (B) after calcination in H_2 at 500°C, and (C) S-1 calcined in air at 500°C. Additional explanations: in the uncalcined catalyst (that is, graph A), a pronounced weight loss and accompanying two differential thermal signals at 350–400°C were observed. These are attributable to the decomposition of tetrapropylammonium cations (TPA^+), ethylenediamine, and ethylenediamine complexes. By contrast, Pt1Cu1@S-1 obtained after H_2 reduction at 500 °C (that is, graph B) showed no such feature, indicating that calcination in H_2 at 500 °C effectively removes nearly all TPA^+ and ethylenediamine species. Slightly, weight loss (0.66 mg responsible for 6.6 wt% sample) was observed for calcined Pt1Cu1@S-1. Note that the Pt1Cu1@S-1 sample was exposed to ambient air prior to the TG–DTA measurement. Therefore, a weight loss attributable to the temperature-induced desorption of water trapped within the zeolite pores formed by the removal of organic species, along with the corresponding variations in the DTA signal, is observable. To substantiate it, we also performed TG-DTA for S-1 pre-calcined under air at 500 °C (typical calcination conditions for removal of organic compounds that occupy the zeolite pores). The obtained datum is responsible for graph C. The TG and DTA curves were similar to those of Pt1Cu1@S-1 pre-calcined in H_2 at 500 °C, verifying the validity of our claim. Accordingly, we can conclude that calcination in H_2 at 500 °C enables the removal of nearly all TPA^+ and ethylenediamine species that occupy the zeolite pores.

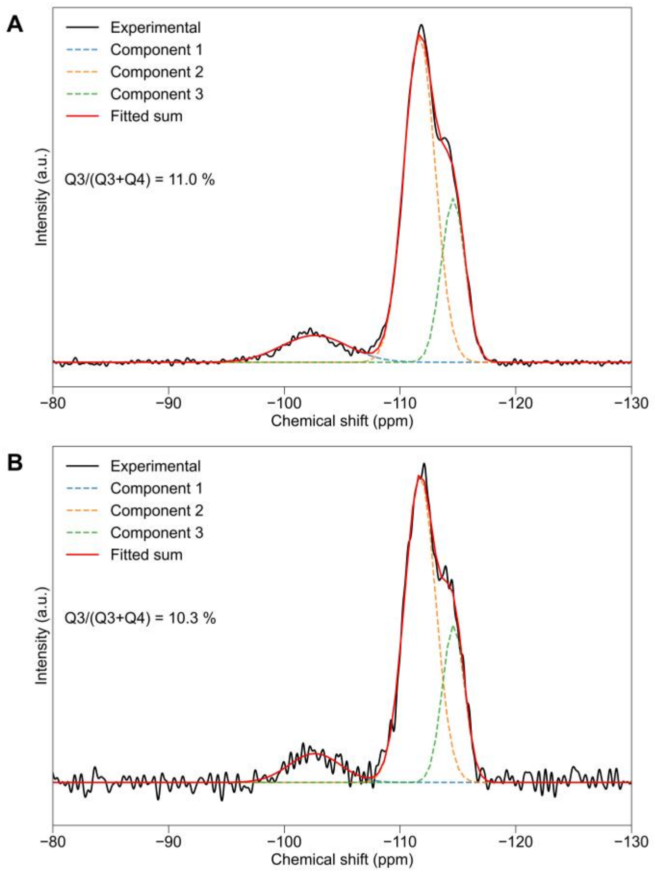

Figure S2. ^{29}Si MAS NMR spectra for Pt1Cu1@S-1 before and after calcination in H_2 at 500°C.

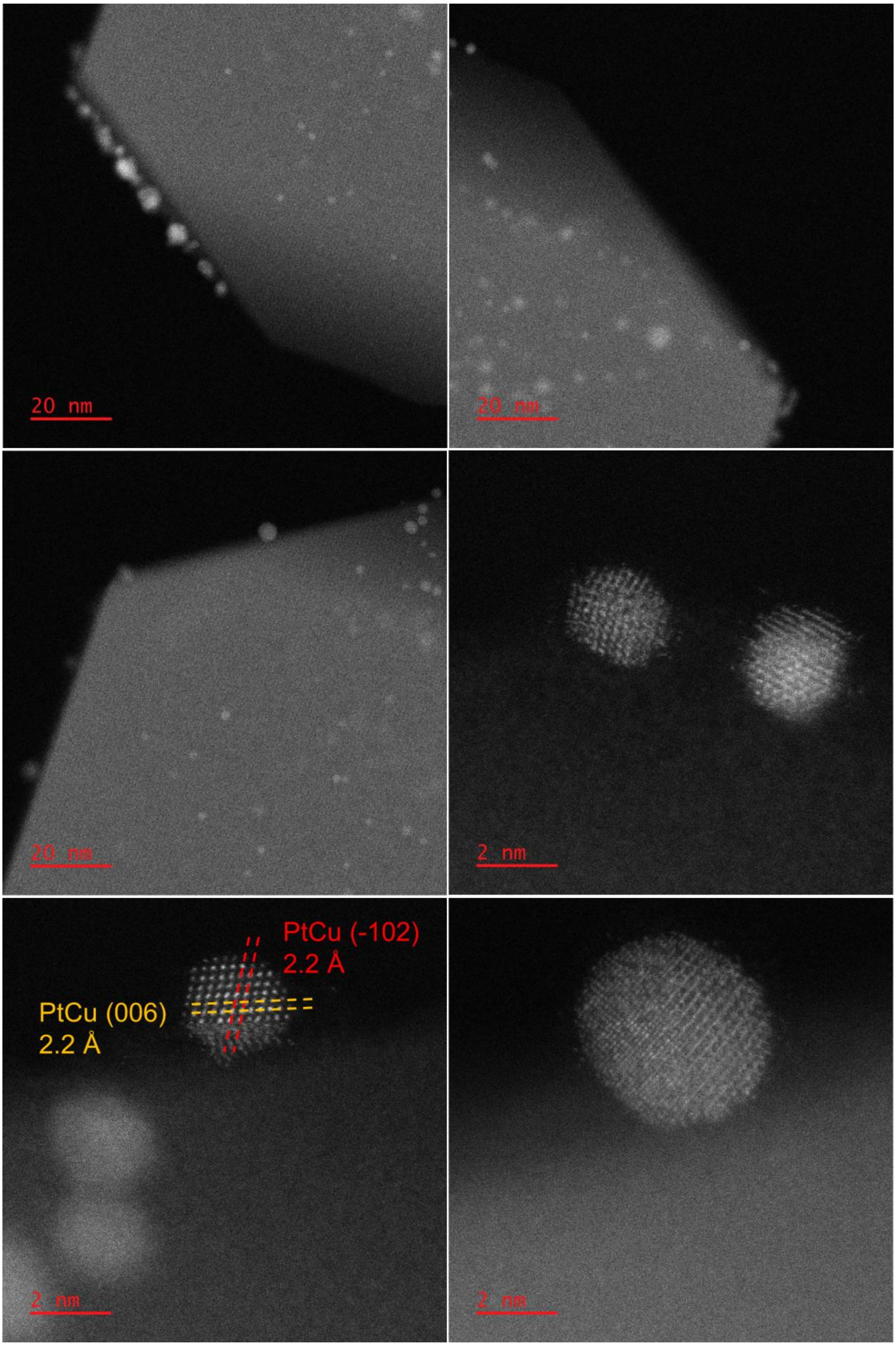

Figure S3. Additional HAADDF-STEM images of Pt1Cu1/S-1.

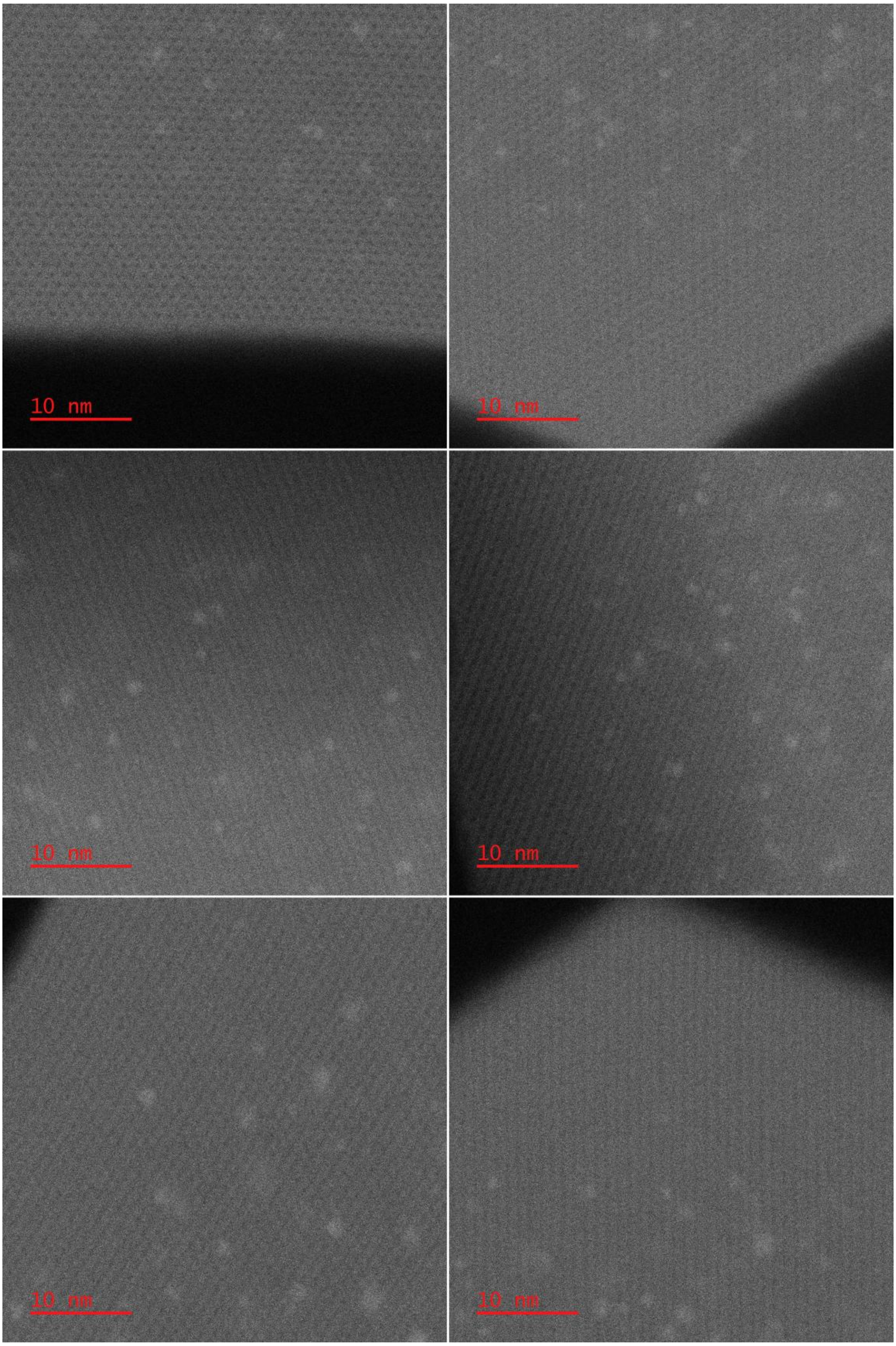

Figure S4. Additional HAADDF-STEM images of Pt1Cu1@S-1.

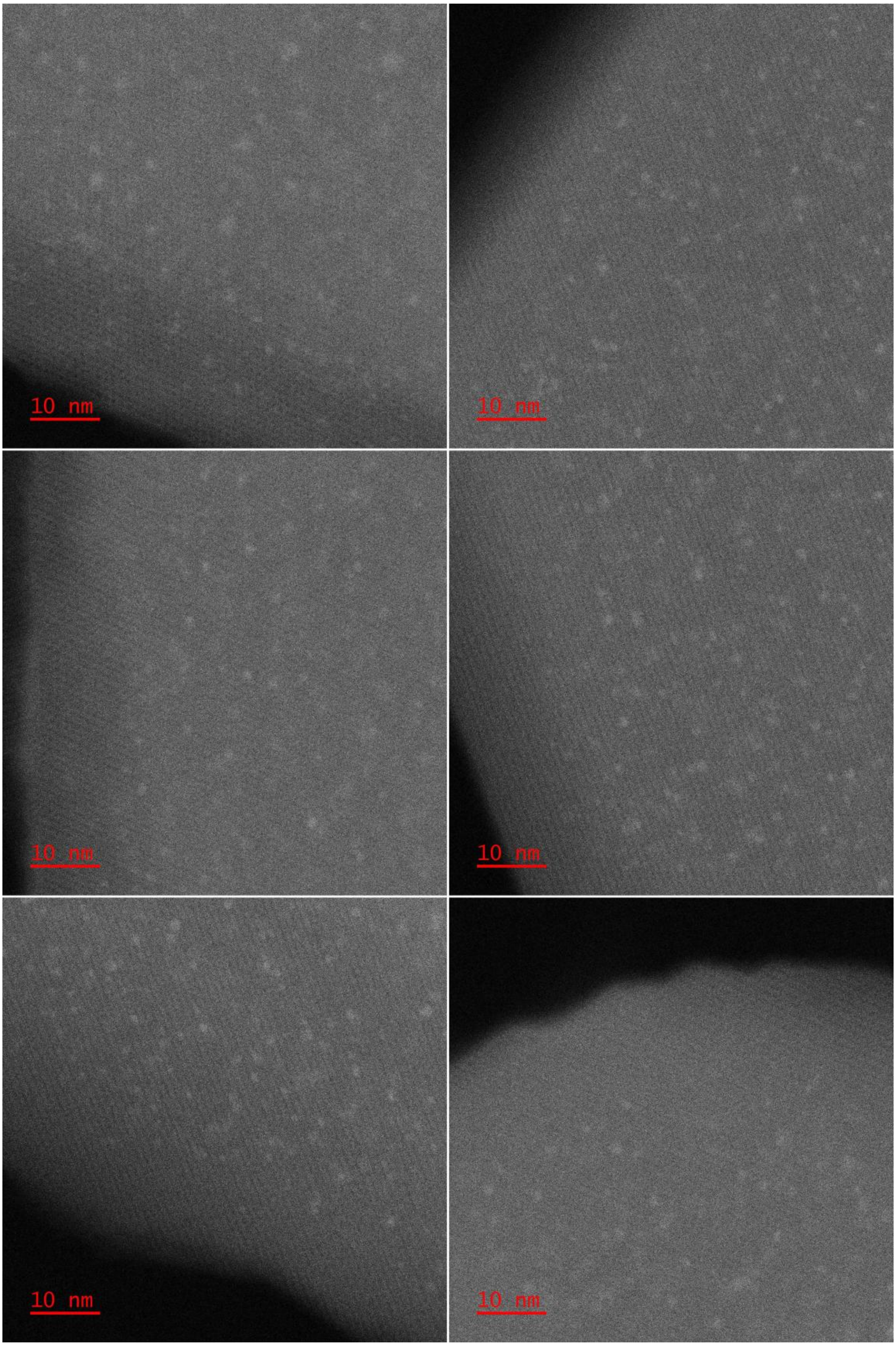

Figure S5. Additional HAADDF-STEM images of Pt@S-1.

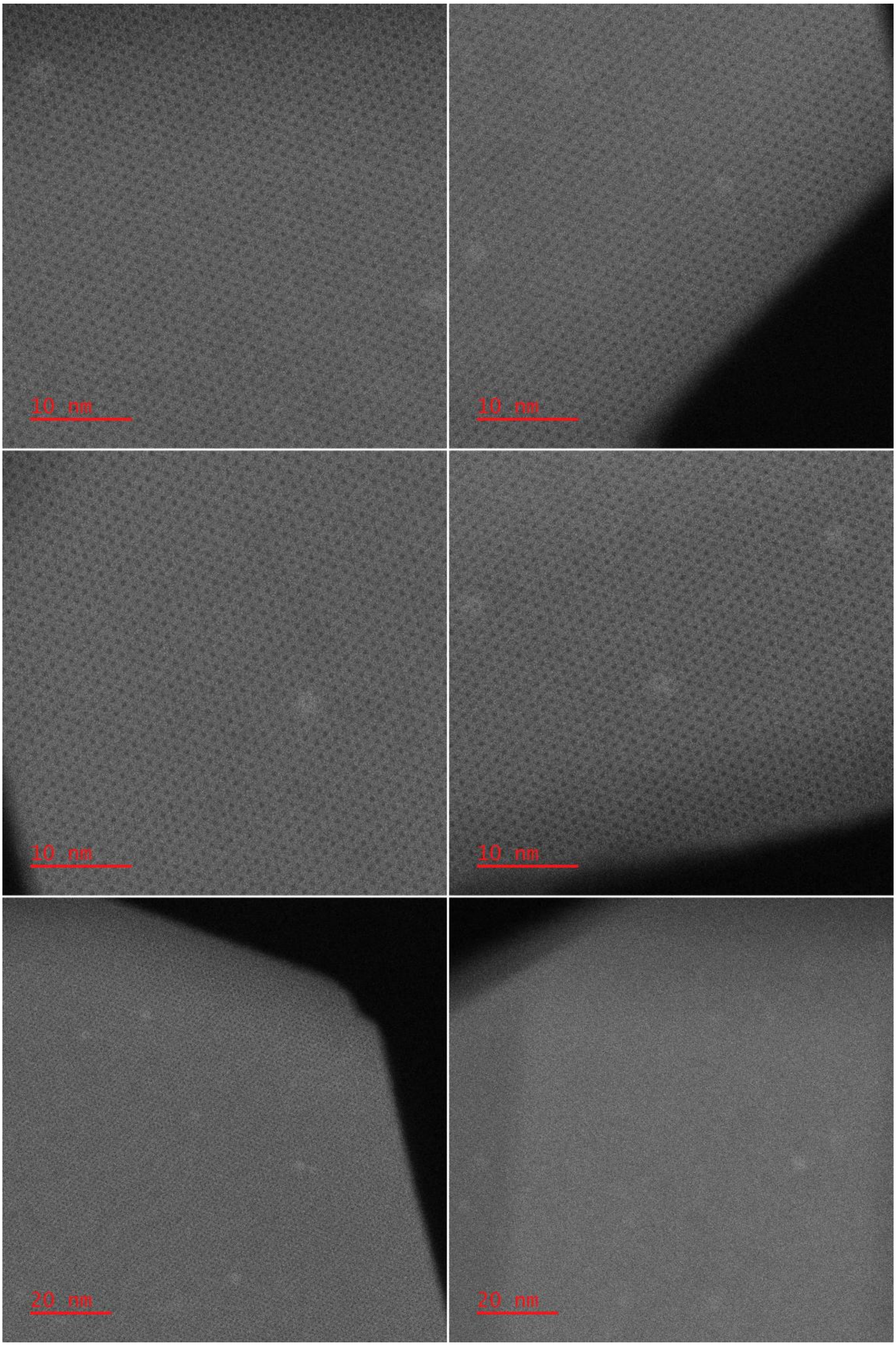

Figure S6. Additional HAADDF-STEM images of Cu@S-1.

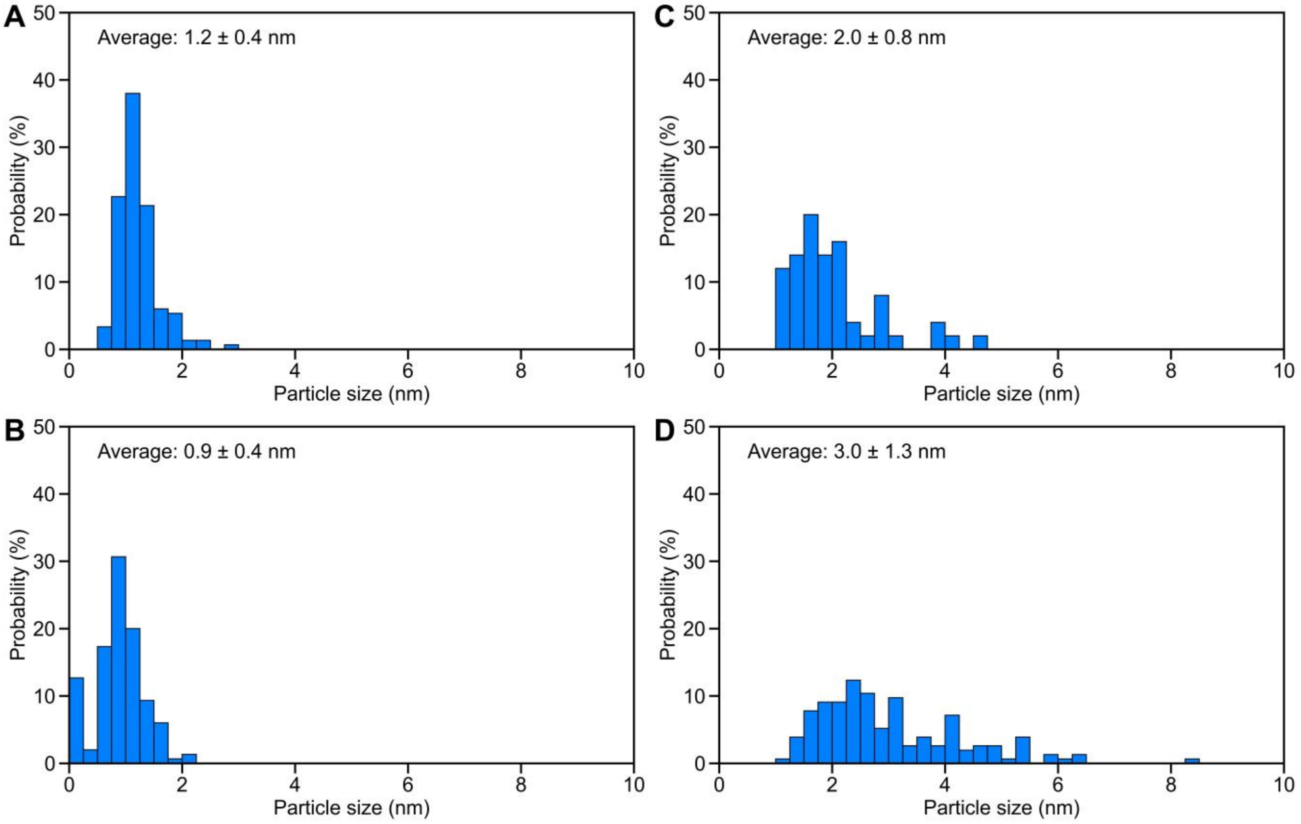

Figure S7. Size distributions of nanoparticles observed by HAADF-STEM: (A) Pt1Cu1@S-1; (B) Pt@S-1; (C) Cu@S-1; (D) Pt1Cu1/S-1.

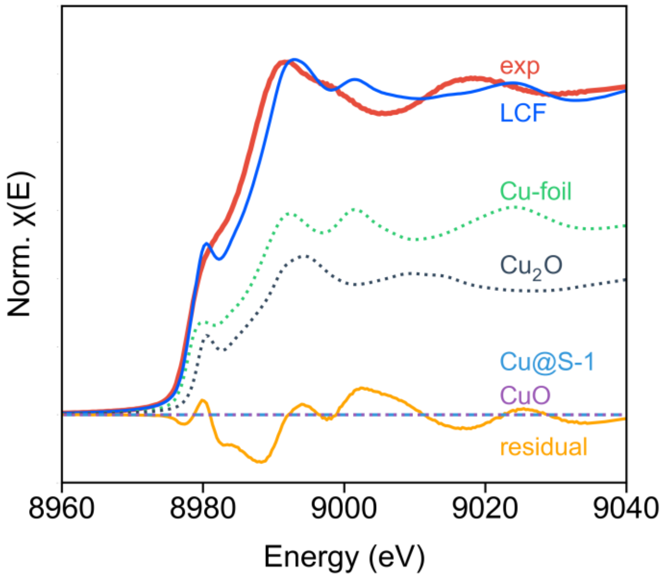

Figure S8. Linear combination fitting of Cu K-edge XANES spectrum for Pt1Cu1@S-1.

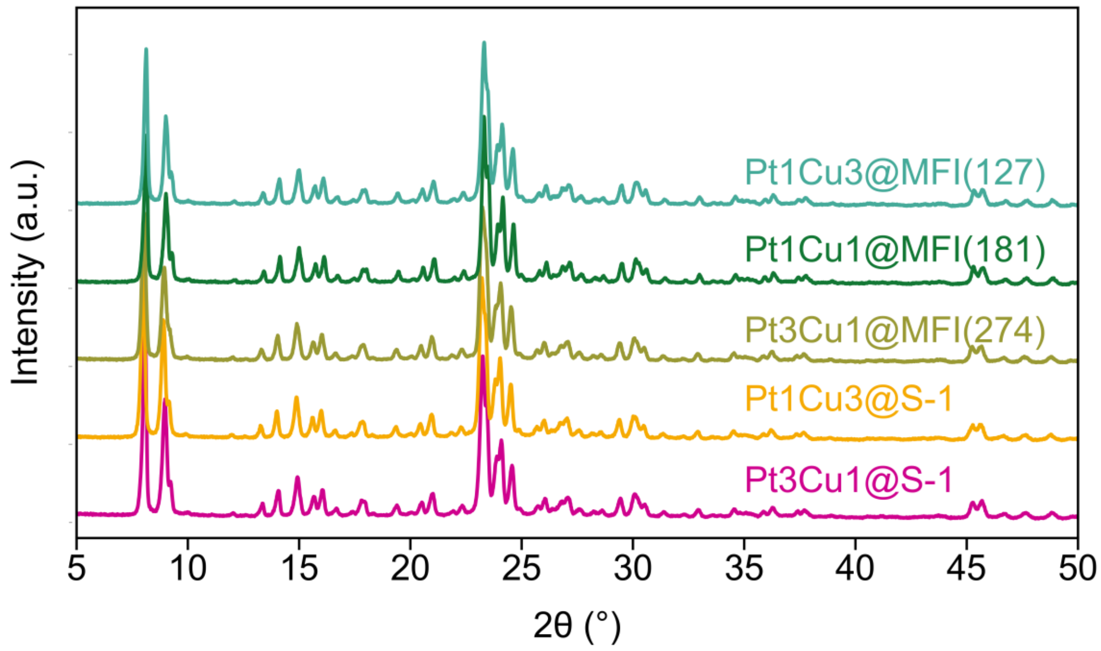

Figure S9. XRD profiles of PtCu@S-1 with different Cu/Pt molar ratios and Pt3Cu1@MFI with different Si/Al ratios.

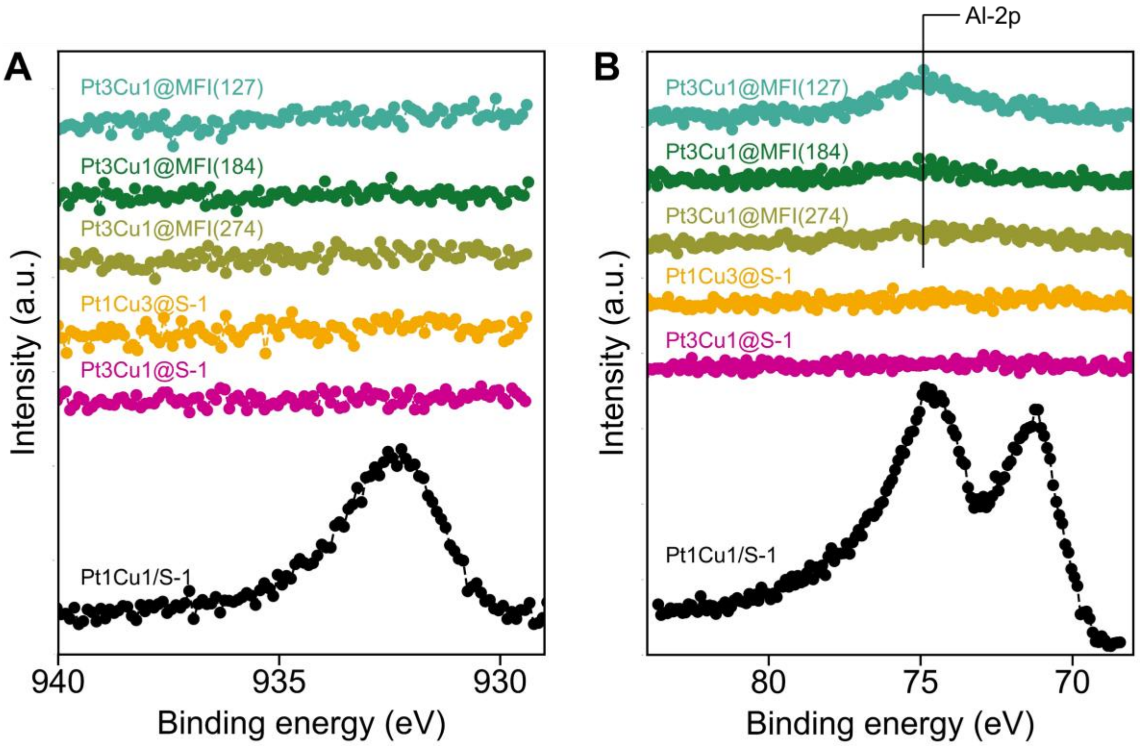

Figure S10. (A) Cu-2p and (B) Pt-4f XPS spectra for PtCu@S-1 with different Cu/Pt molar ratios and Pt3Cu1@MFI with different Si/Al ratios.

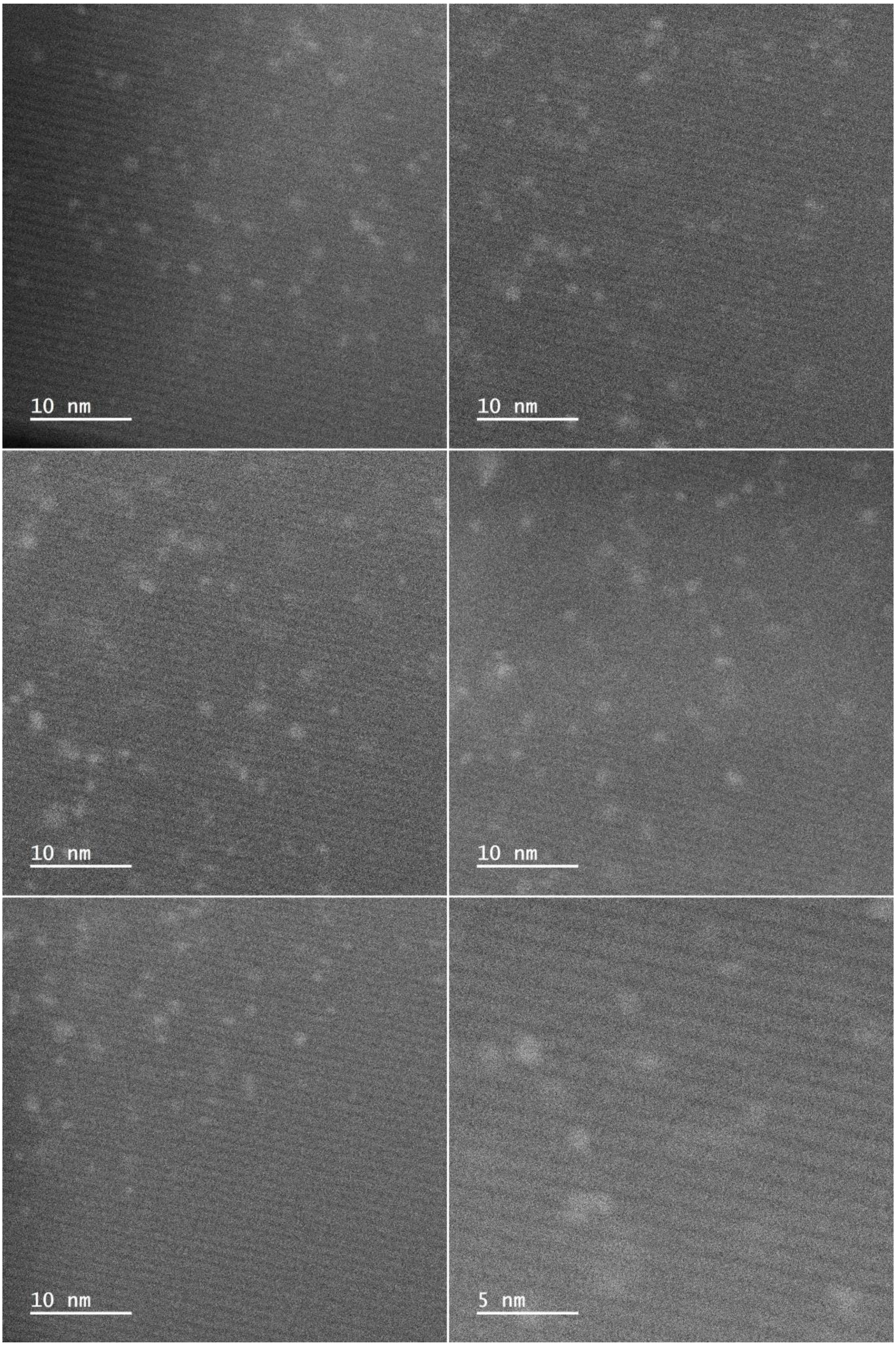

Figure S11. HAADDF-STEM images of Pt3Cu1@S-1.

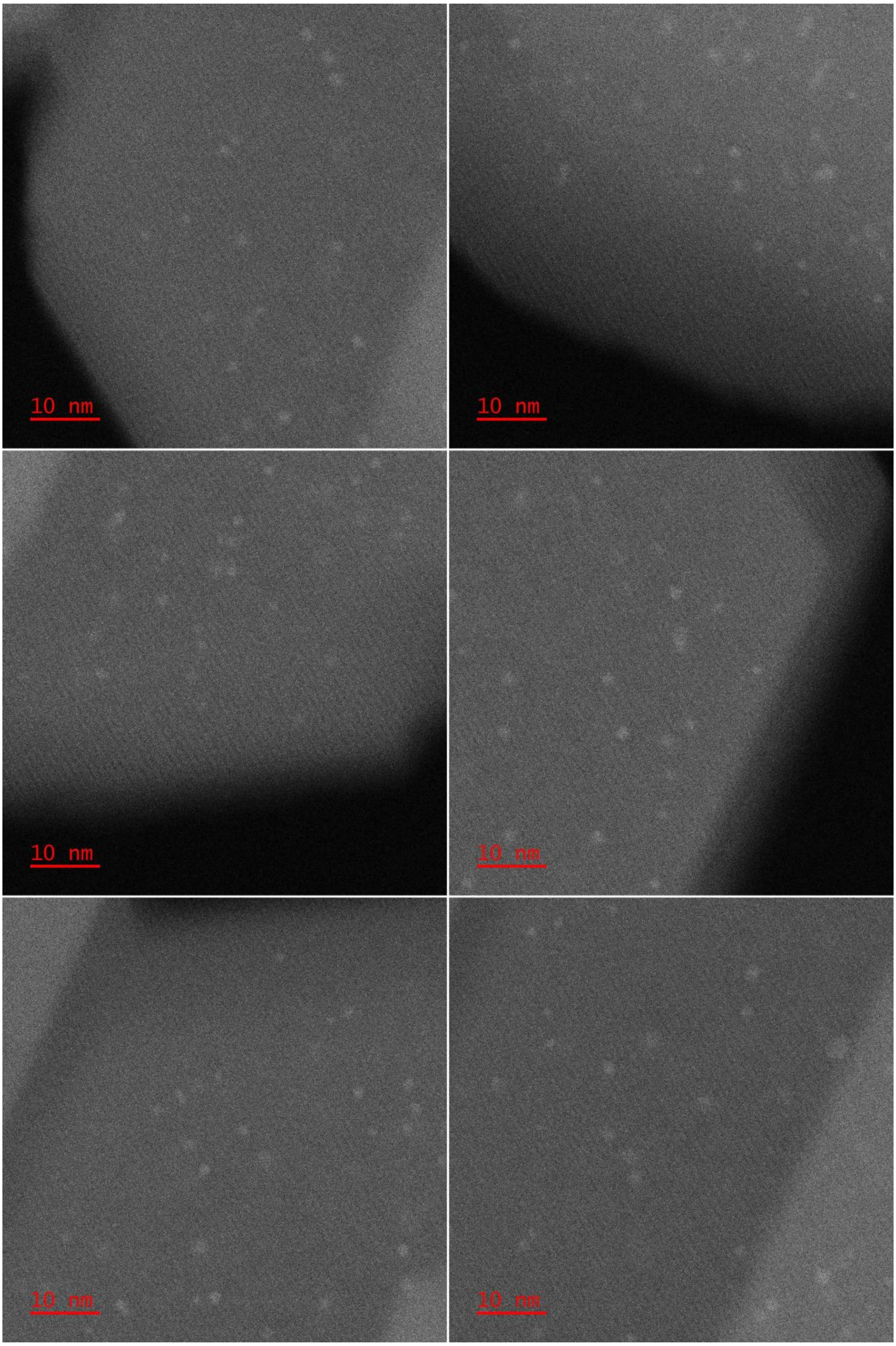

Figure S12. HAADDF-STEM images of Pt1Cu3@S-1.

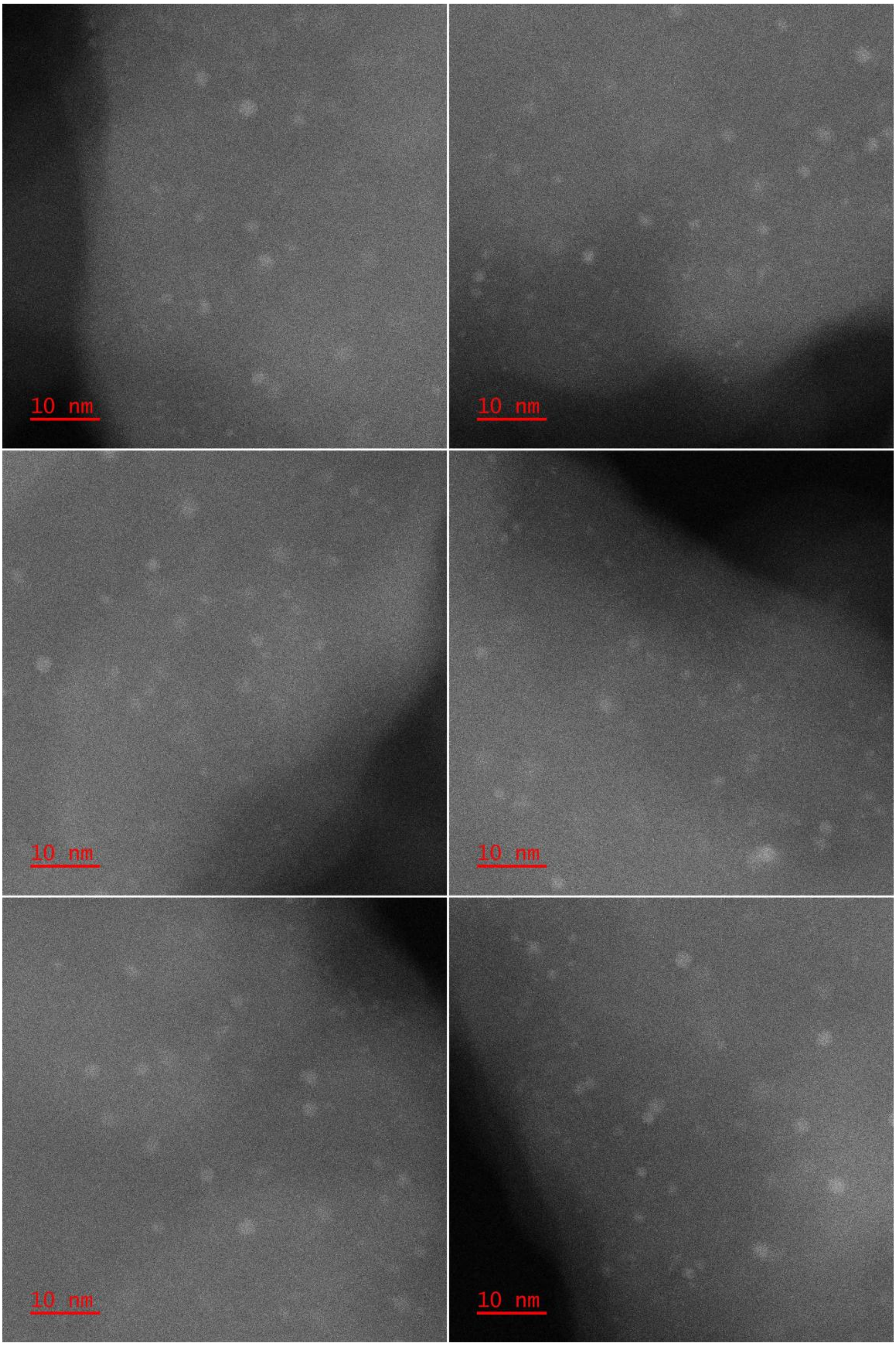

Figure S13. HAADDF-STEM images of Pt3Cu1@MFI(127).

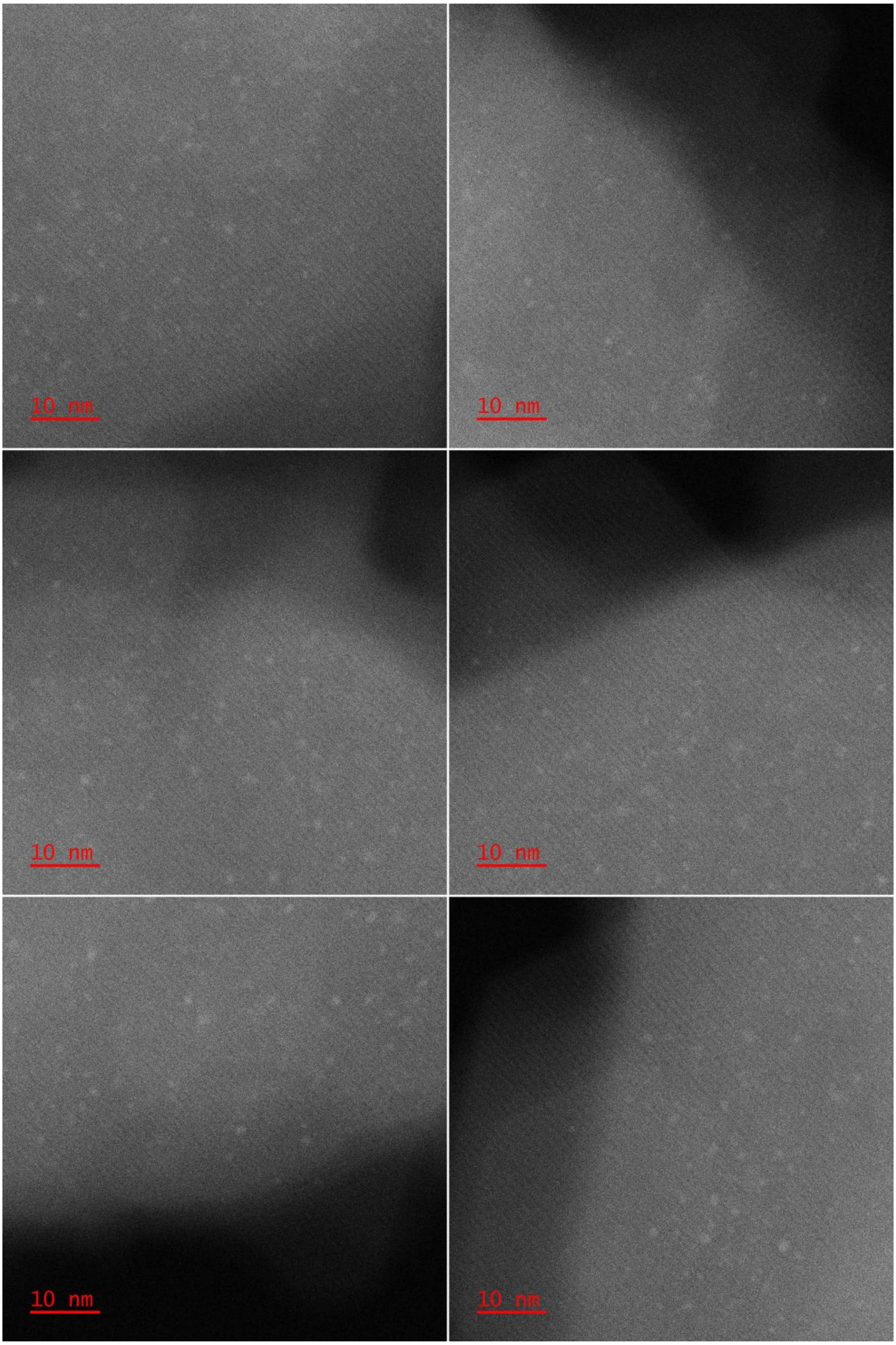

Figure S14. HAADDF-STEM images of Pt3Cu1@MFI(181).

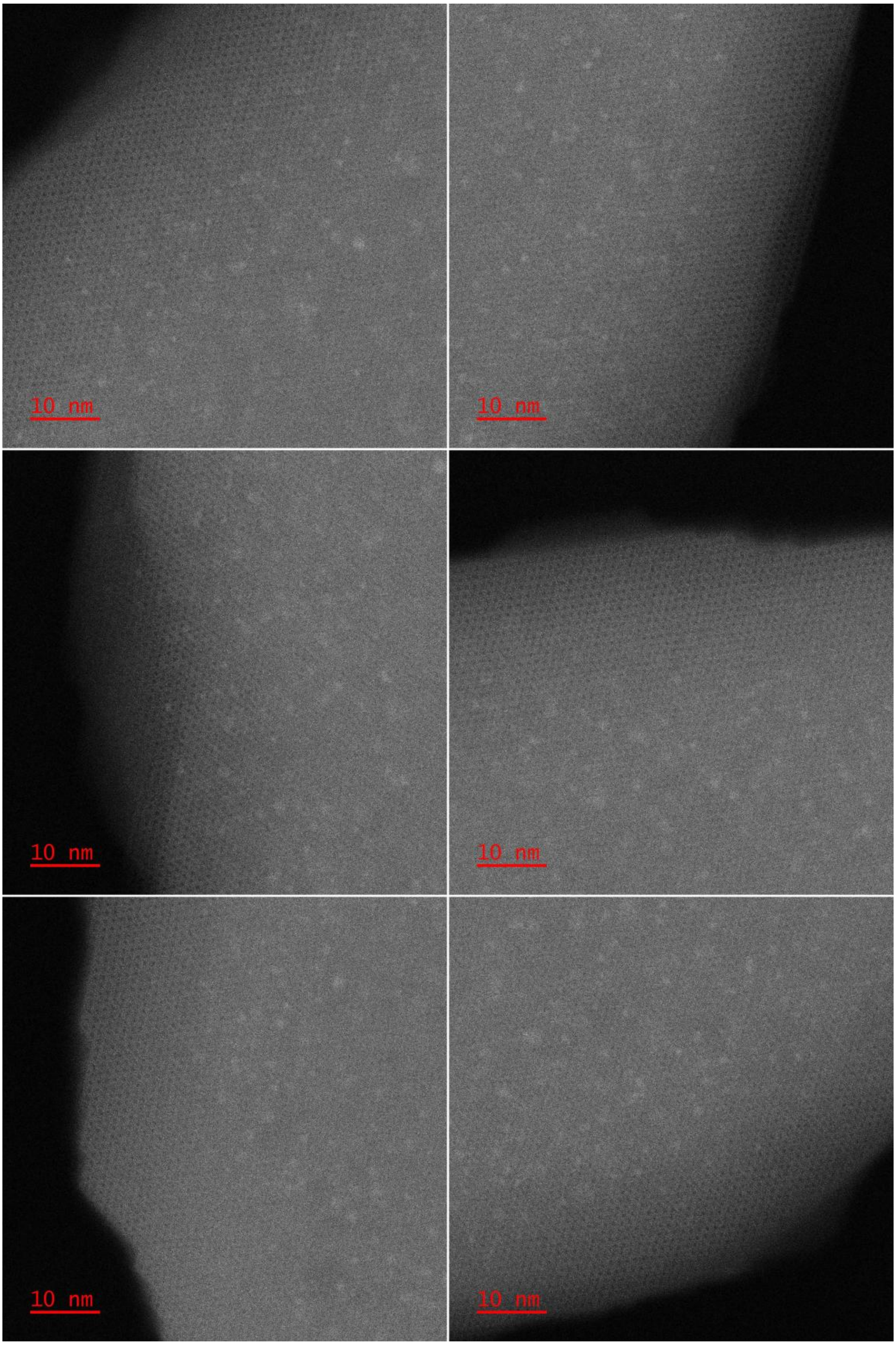

Figure S15. HAADDF-STEM images of Pt3Cu1@MFI(274).

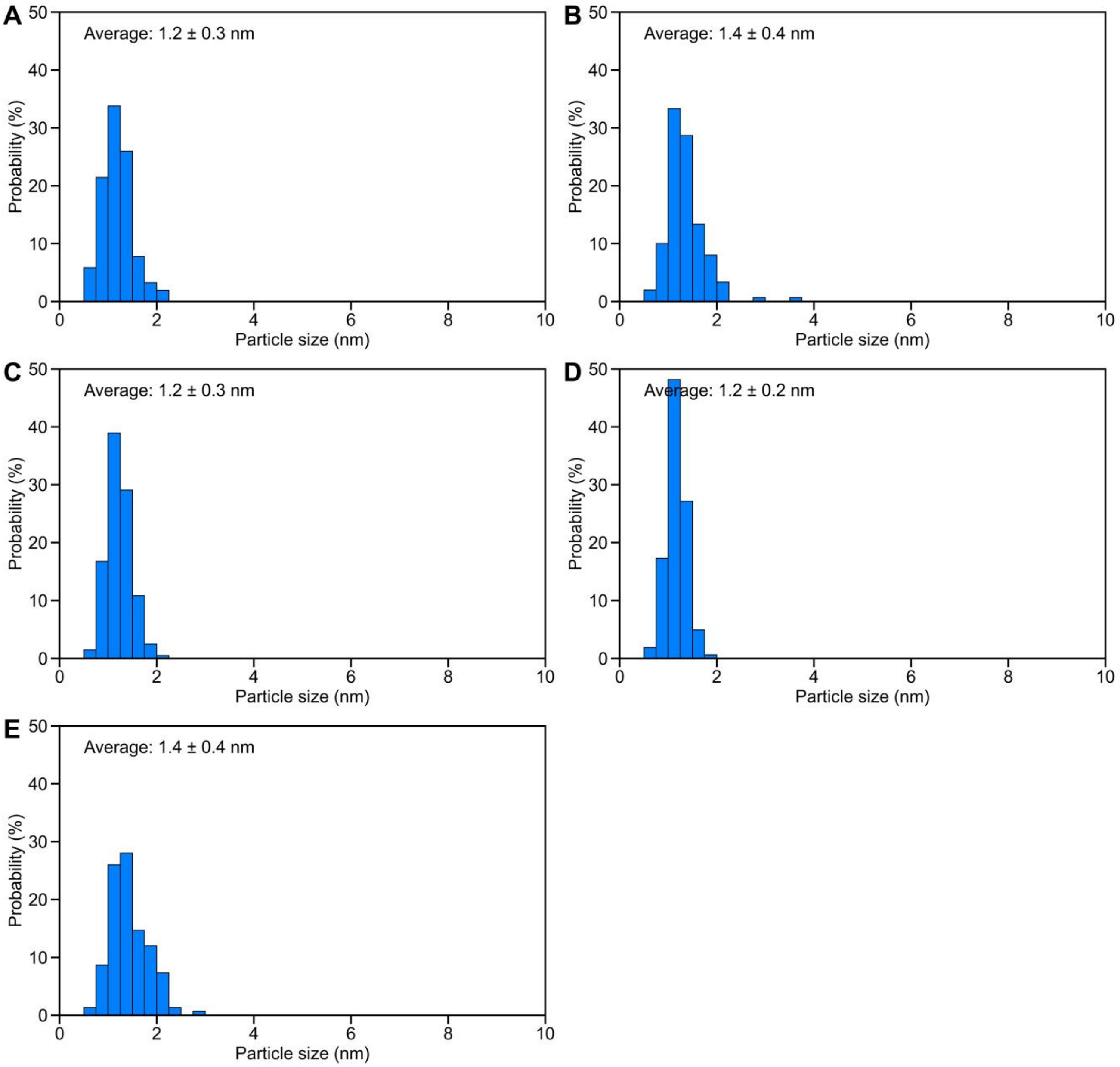

Figure S16. Size distributions of nanoparticles observed by HAADF-STEM: (A) Pt3Cu1@S-1, (B) Pt1Cu3@S-1, (C) Pt3Cu1@MFI(274), (D) Pt3Cu1@MFI(181), and (E) Pt3Cu1@MFI(127).

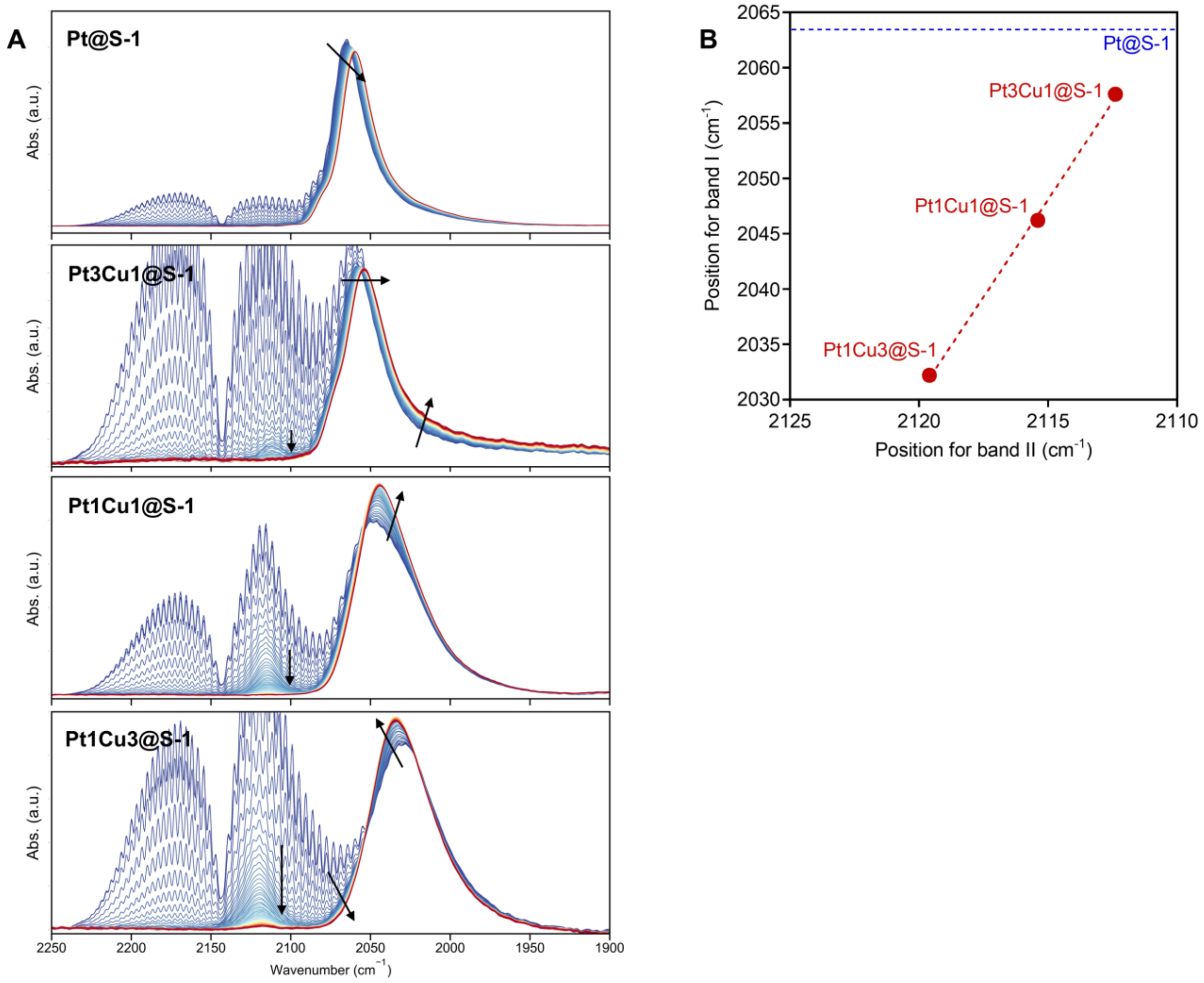

Figure S17. (A) Comparison of time-resolved CO-FTIR spectra of Pt@S-1, Pt3Cu1@S-1, Pt1Cu1@S-1, and Pt1Cu3@S-1 during the Ar-purge process after CO adsorption. The characteristics of the change in band intensity and position over time are indicated by arrows. (B) A relationship between the band positions I and II observed for the respective catalysts.

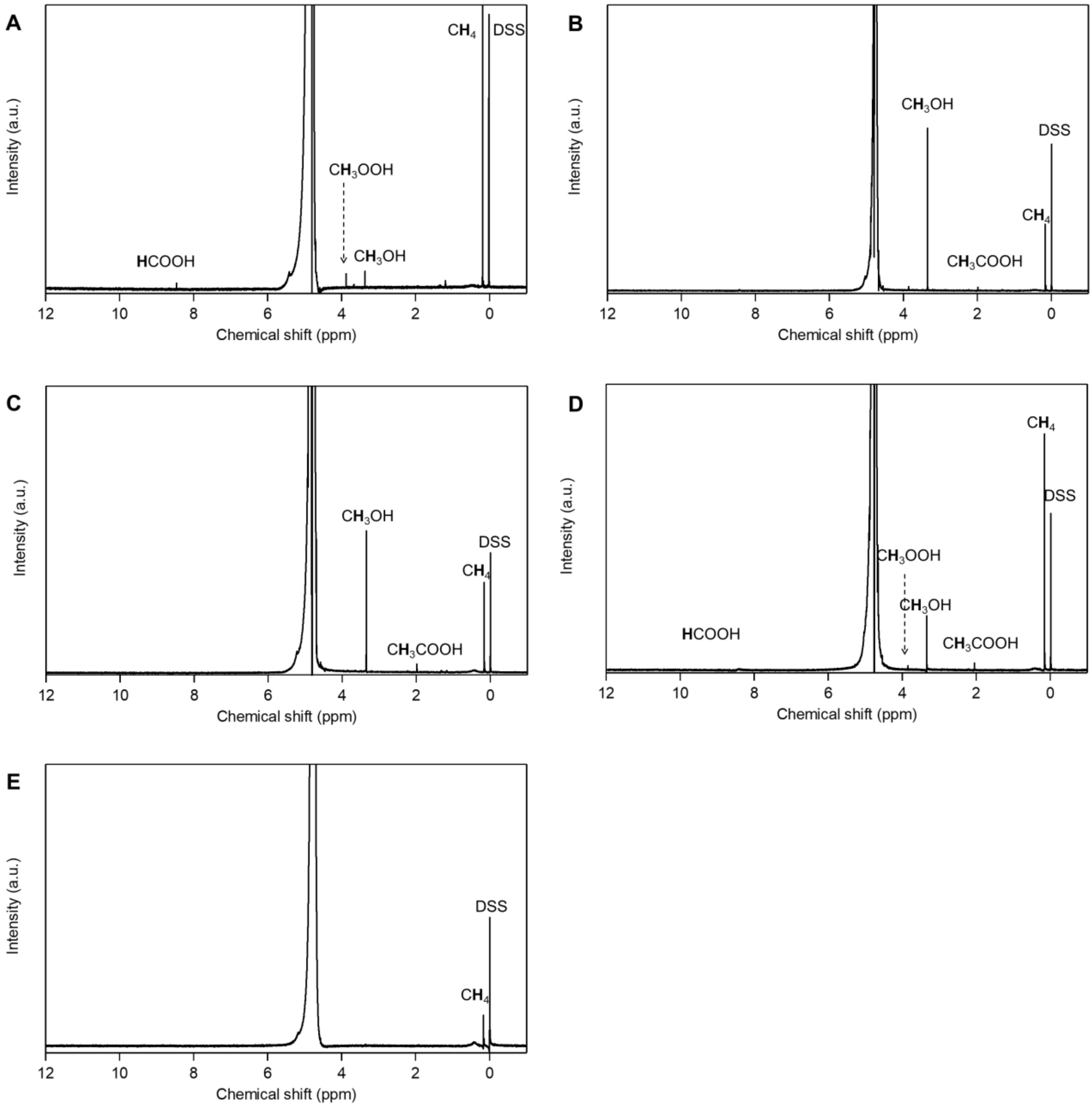

Figure S18. Representative ^{1}H NMR spectra of the products in the CO-assisted oxidation of CH_4 over (A) Pt@S-1, (B) Pt3Cu1@S-1, (C) Pt1Cu1@S-1, (D) Pt1Cu3@S-1, and Cu@S-1. Reaction conditions: catalyst loading, 5 mg; solvent, 15 ml of H_2O; reaction gas, 20 bar CH_4 + 5 bar CO + 3 bar O_2; temperature, 150 ˚C; reaction time, 1 h.

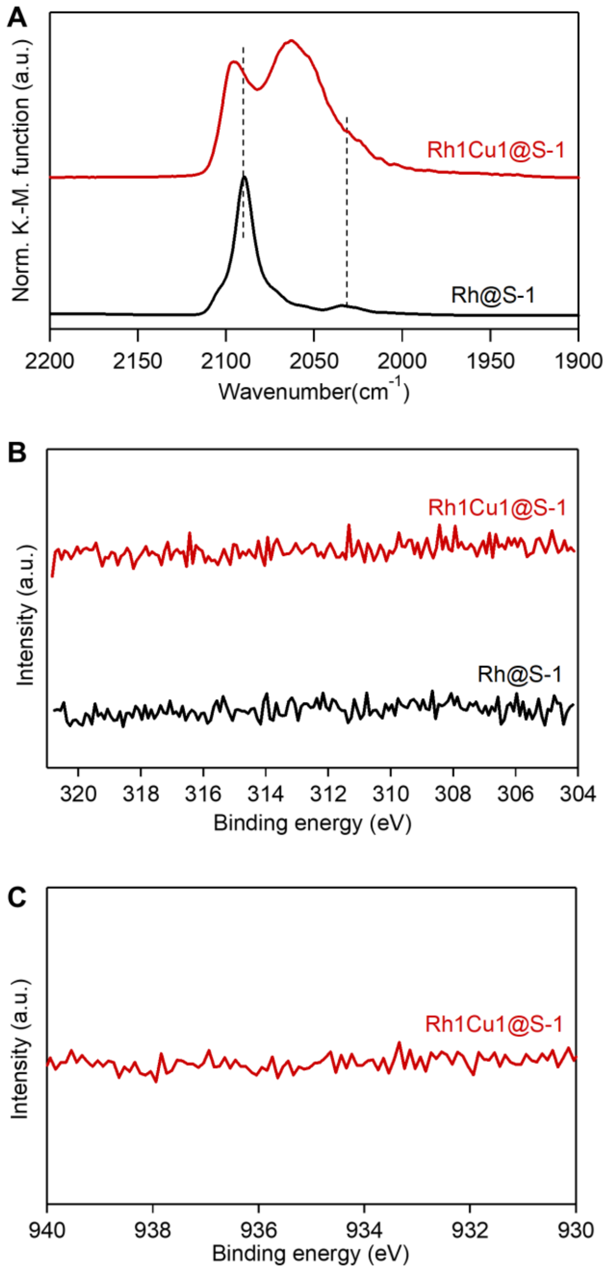

Figure S19. (A) CO-DRIFT spectra and (B) Rh-3d XPS spectra for Rh@S-1 and Rh1Cu1@S-1. (C) Cu-2p XPS spectrum for Rh1Cu1@S-1.

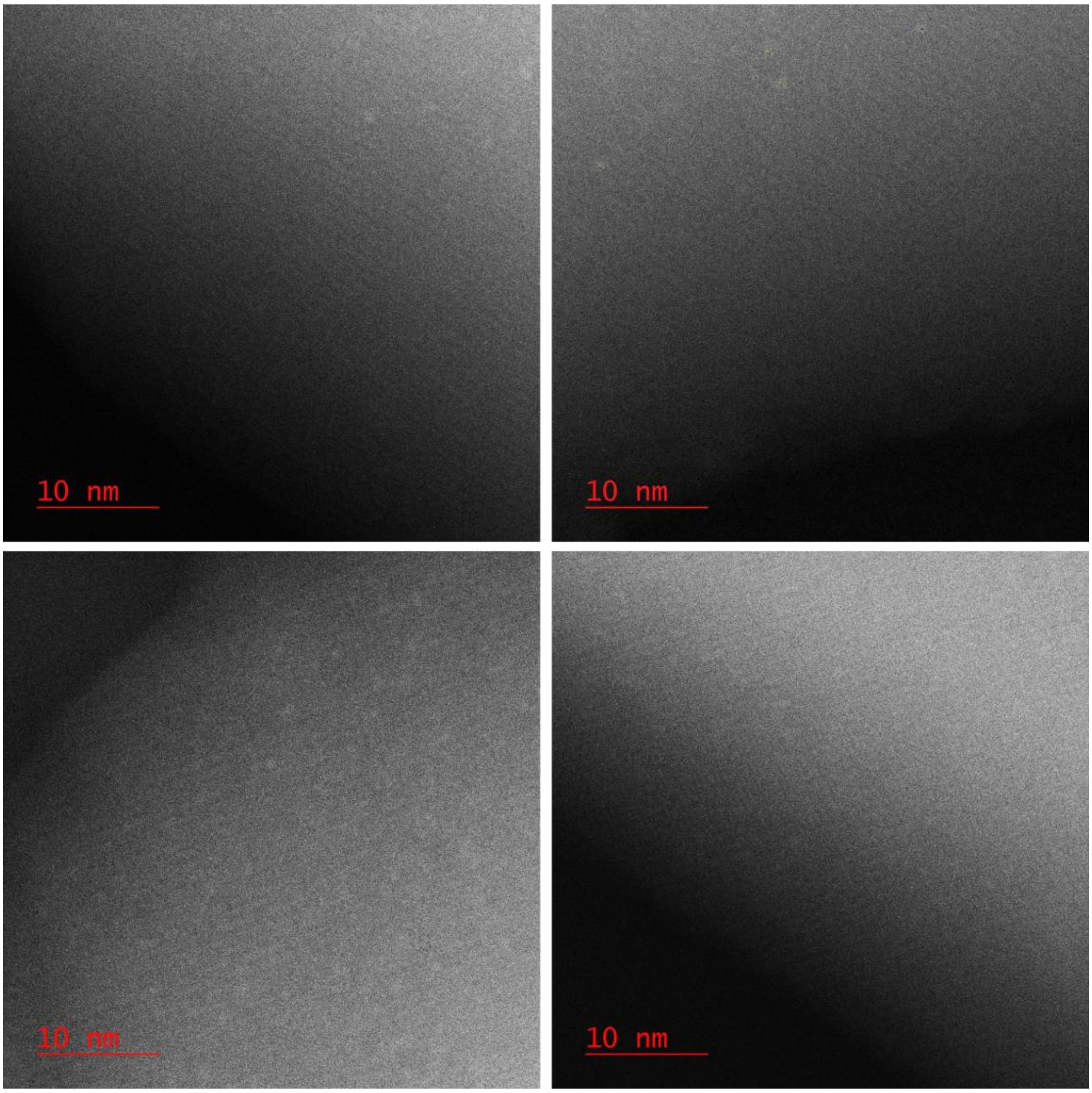

Figure S20. HAADDF-STEM images of Rh1Cu1@S-1.

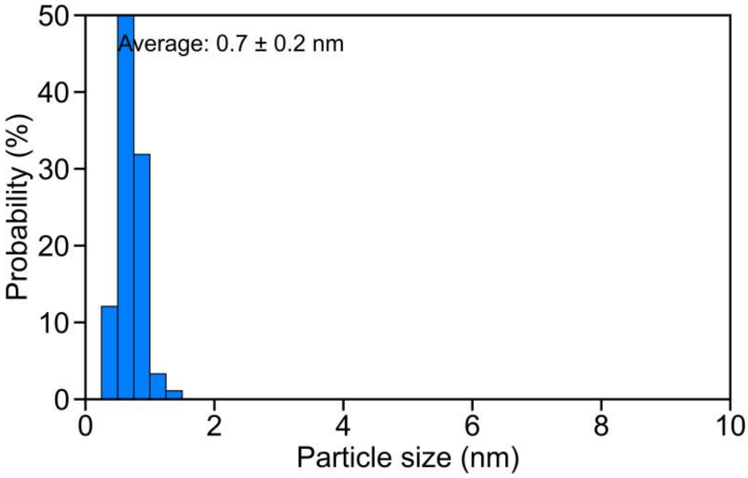

Figure S21. The size distribution of nanoparticles observed for Rh1Cu1@S-1.

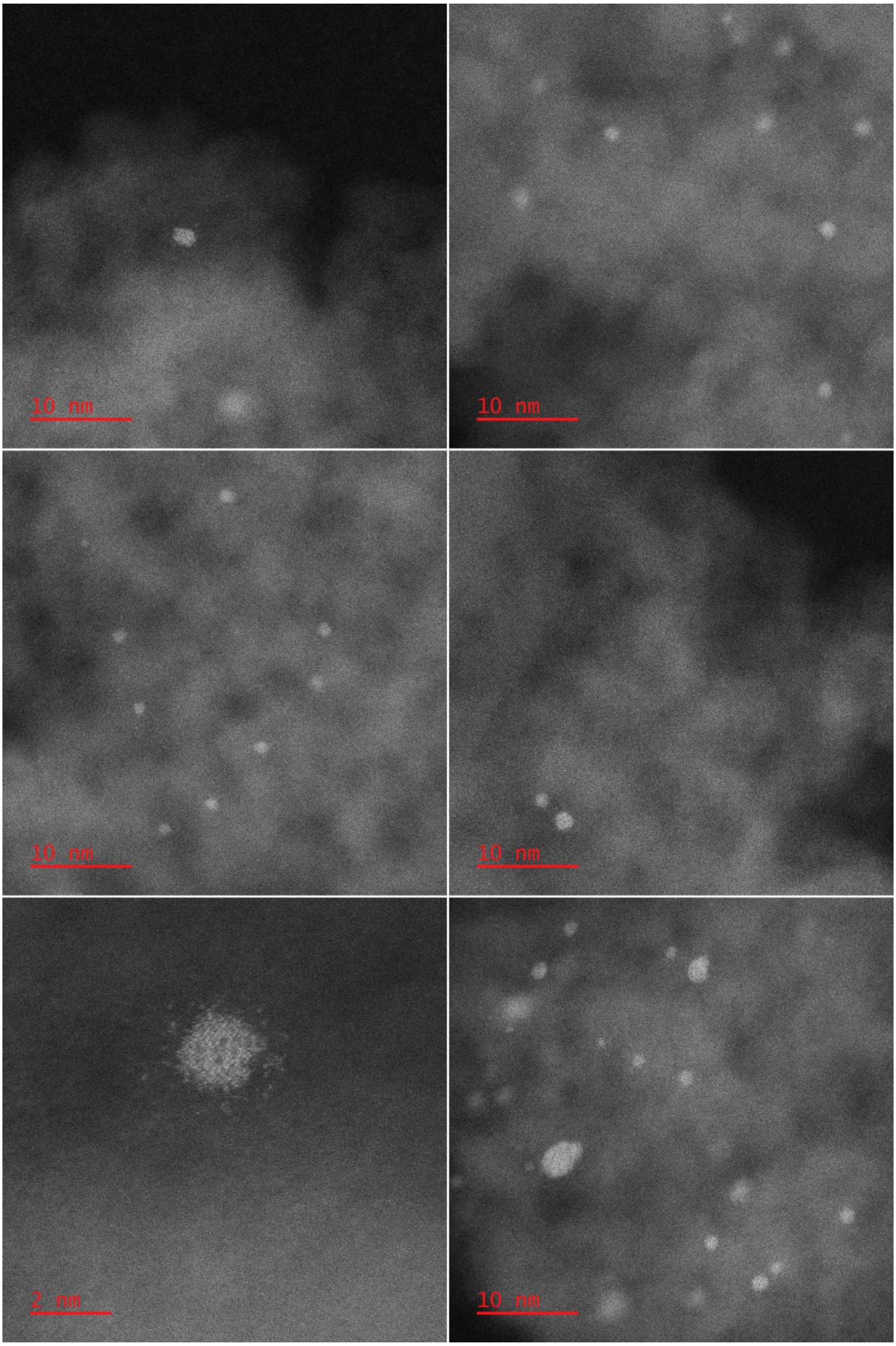

Figure S22. HAADDF-STEM images of Pt1Cu1/SiO_2.

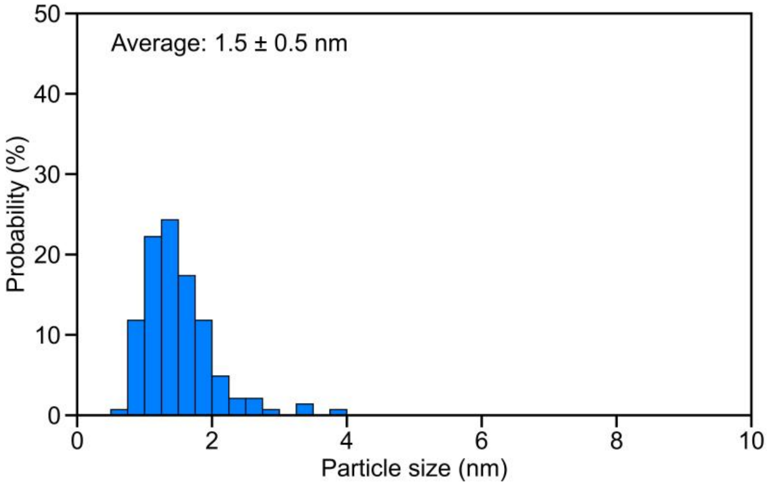

Figure S23. The size distribution of nanoparticles observed for Pt1Cu1/SiO_2.

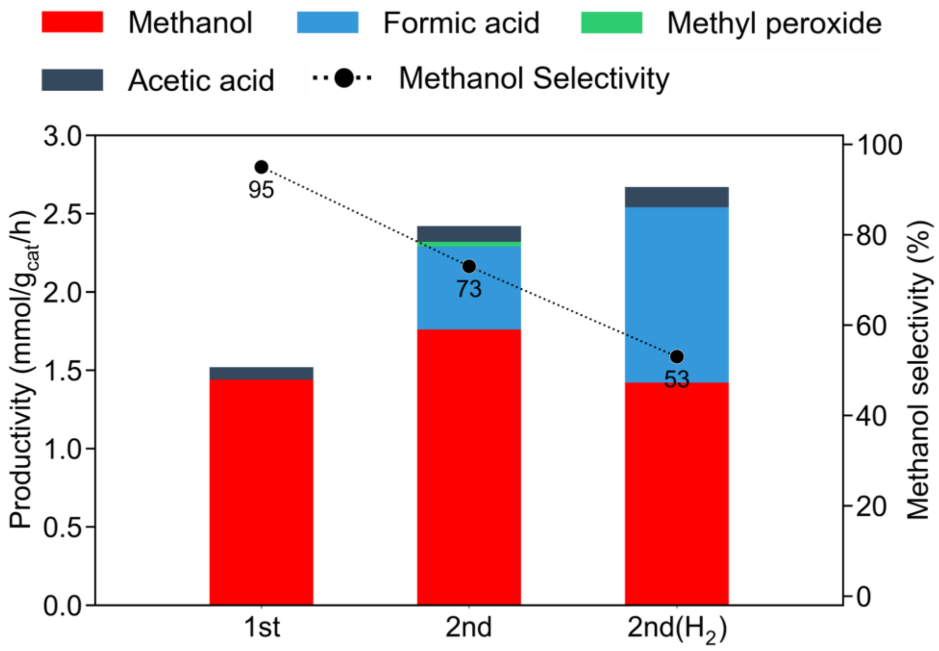

Figure S24. Product distribution and methanol selectivity in recycling tests for CH_4 oxidation over Pt1Cu1@S-1. Reaction conditions: solvent, 15 mL of H_2O; reaction gas, 20 bar CH_4 + 5 bar CO + 3 bar O_2; catalyst loading, 5 mg; reaction temperature, 150 °C; reaction time, 1 h. The first run (1st) used fresh catalyst, the second run (2nd) employed the recovered catalyst without pre-treatment, and 2nd(H_2) corresponds to the recovered catalyst after H_2-reduction pretreatment at 500 °C for 2 h.

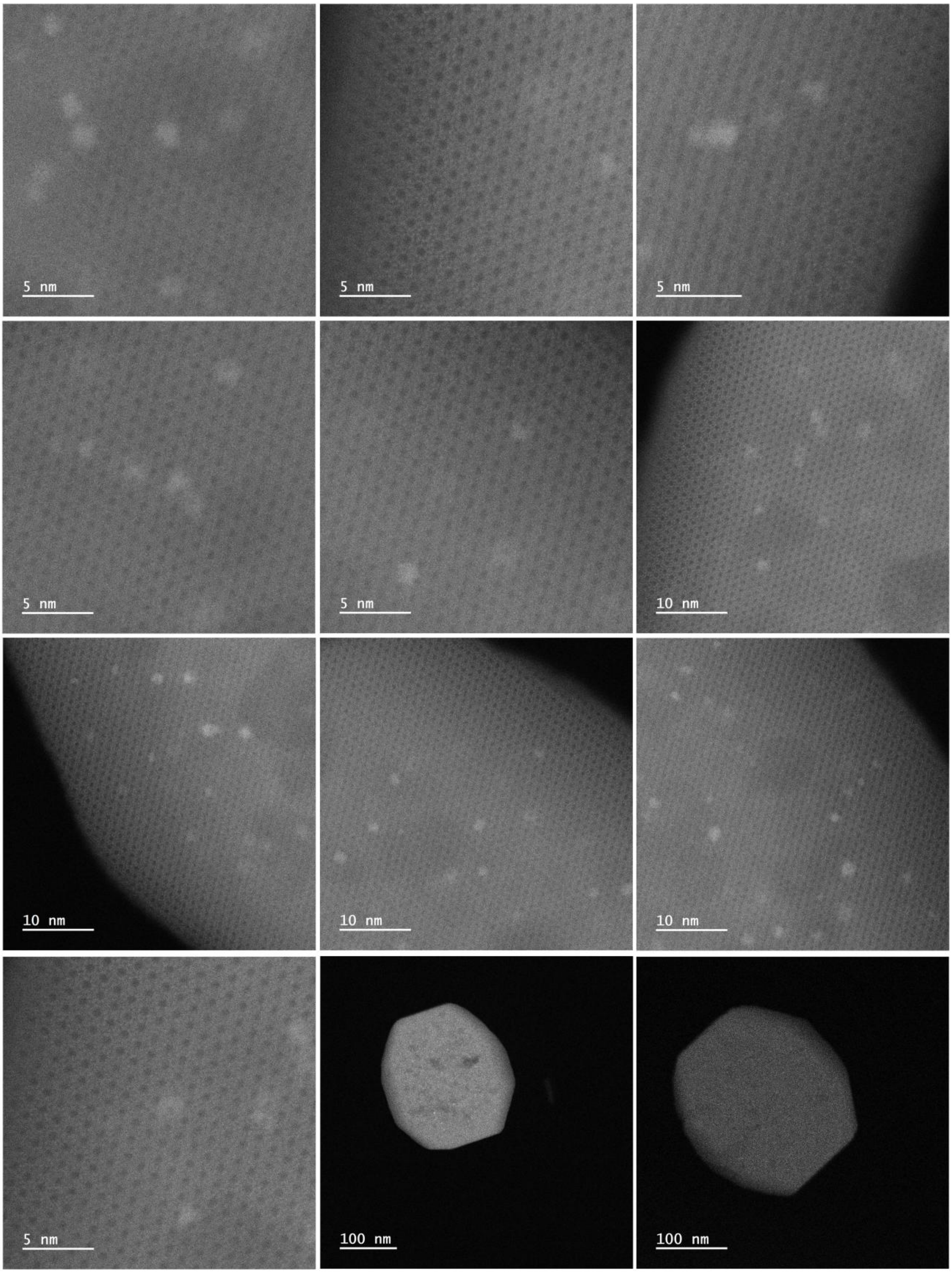

Figure S25. HAADF-STEM images of Pt1Cu1@S-1 after reaction for 1 h.

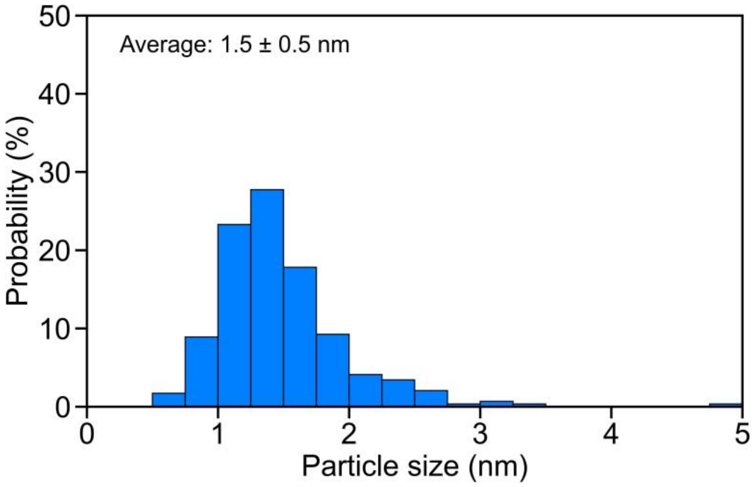

Figure S26. The size distribution of nanoparticles observed for Pt1Cu1@S-1 after reaction for 1h.

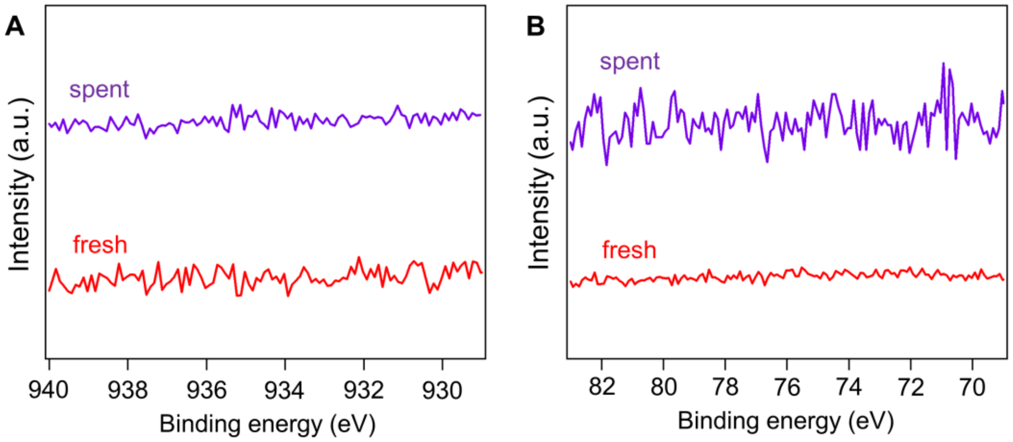

Figure S27. (A) Cu-2p and (B) Pt-4f XPS spectra of Pt1Cu1@S-1 before and after reaction for 1 h.

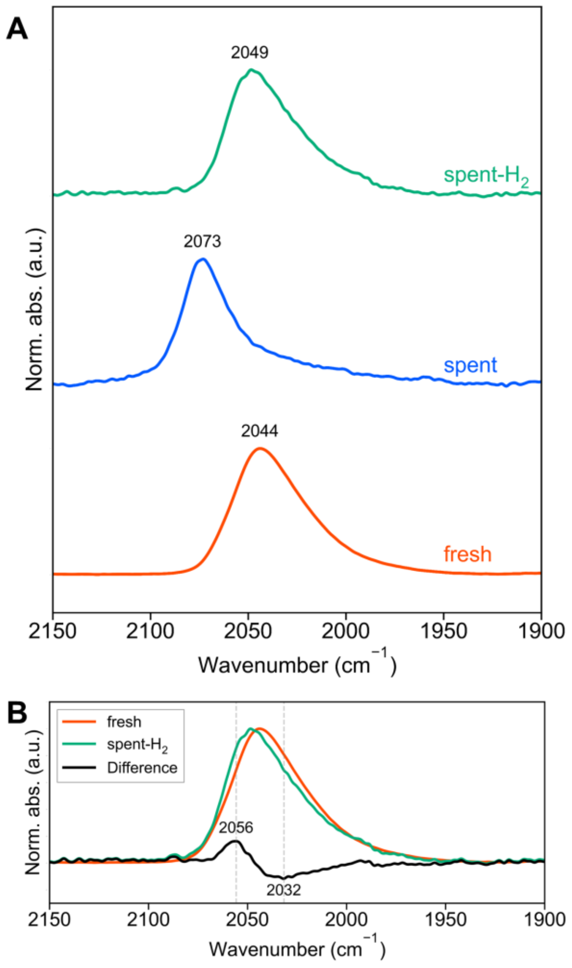

Figure S28. (A) CO-FTIR spectra of Pt1Cu1@S-1: fresh catalyst; spent catalyst without pre-treatment; spent catalyst after H_2-reduction pretreatment at 500 °C for 2 h. (B) Difference spectrum.

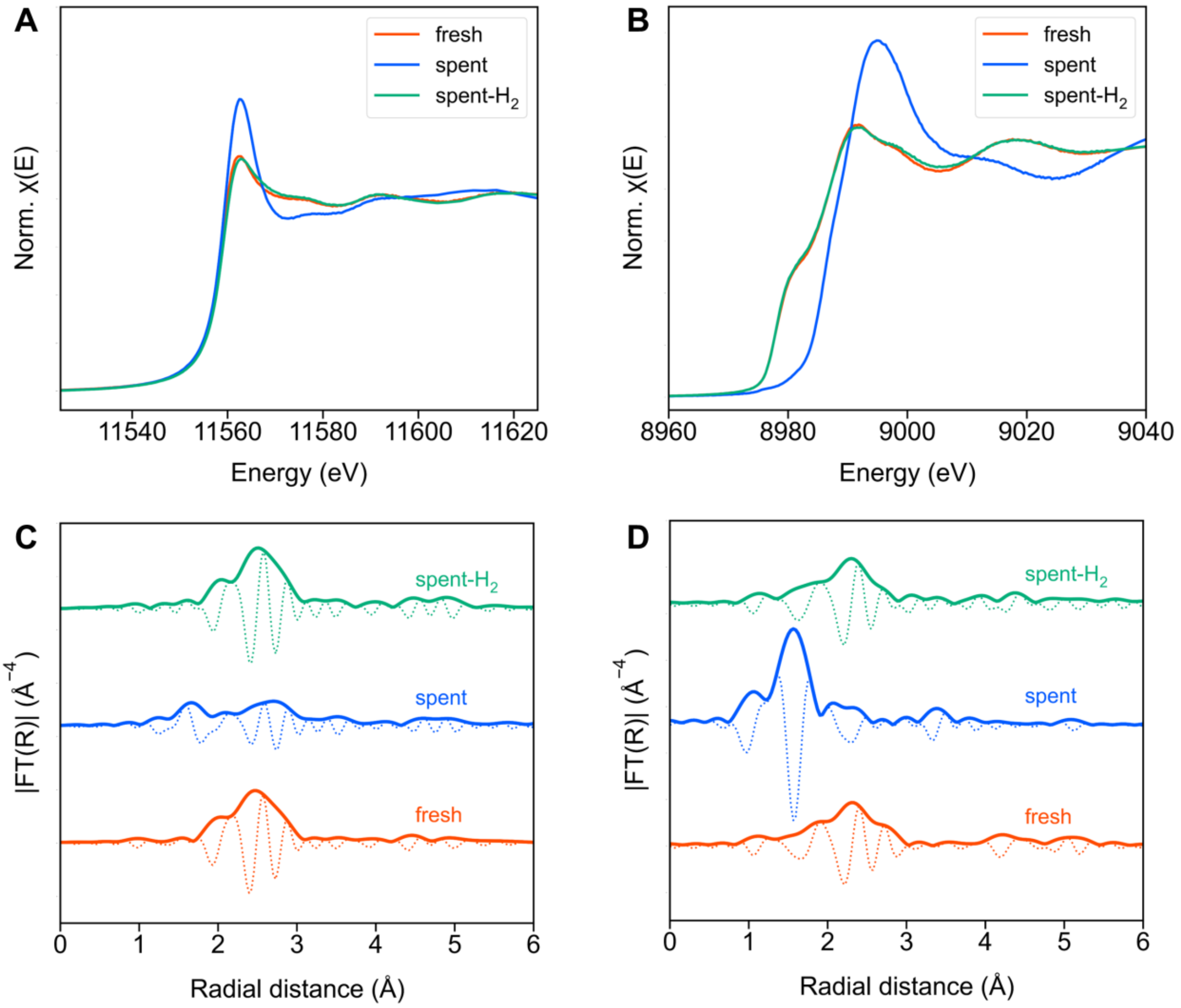

Figure S29. XAFS data for Pt1Cu1@S-1 in three states: fresh, spent (no pretreatment), and spent after H_2-reduction at 500 °C for 2 h. (A, B) Pt L_{III}-edge and Cu K-edge XANES spectra, respectively; (C, D) k^3-weighted FT-EXAFS spectra.

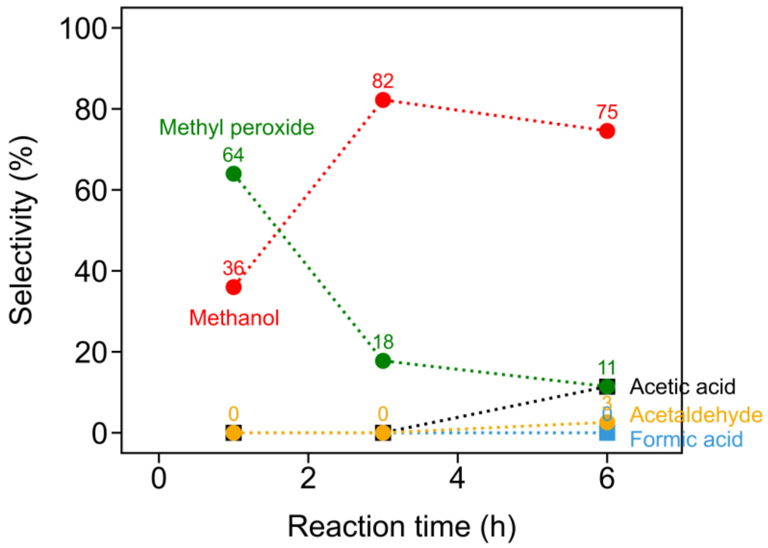

Figure S30. Time-course of selectivity during the low-temperature reaction over Pt1Cu1@S-1. Reaction conditions: catalyst, 5 mg; solvent, 15 ml of H_2O; reaction gas, 20 bar CH_4 + 5 bar CO + 3 bar O_2; reaction temperature, 100 ˚C; reaction time, 1, 3, and 6 h.

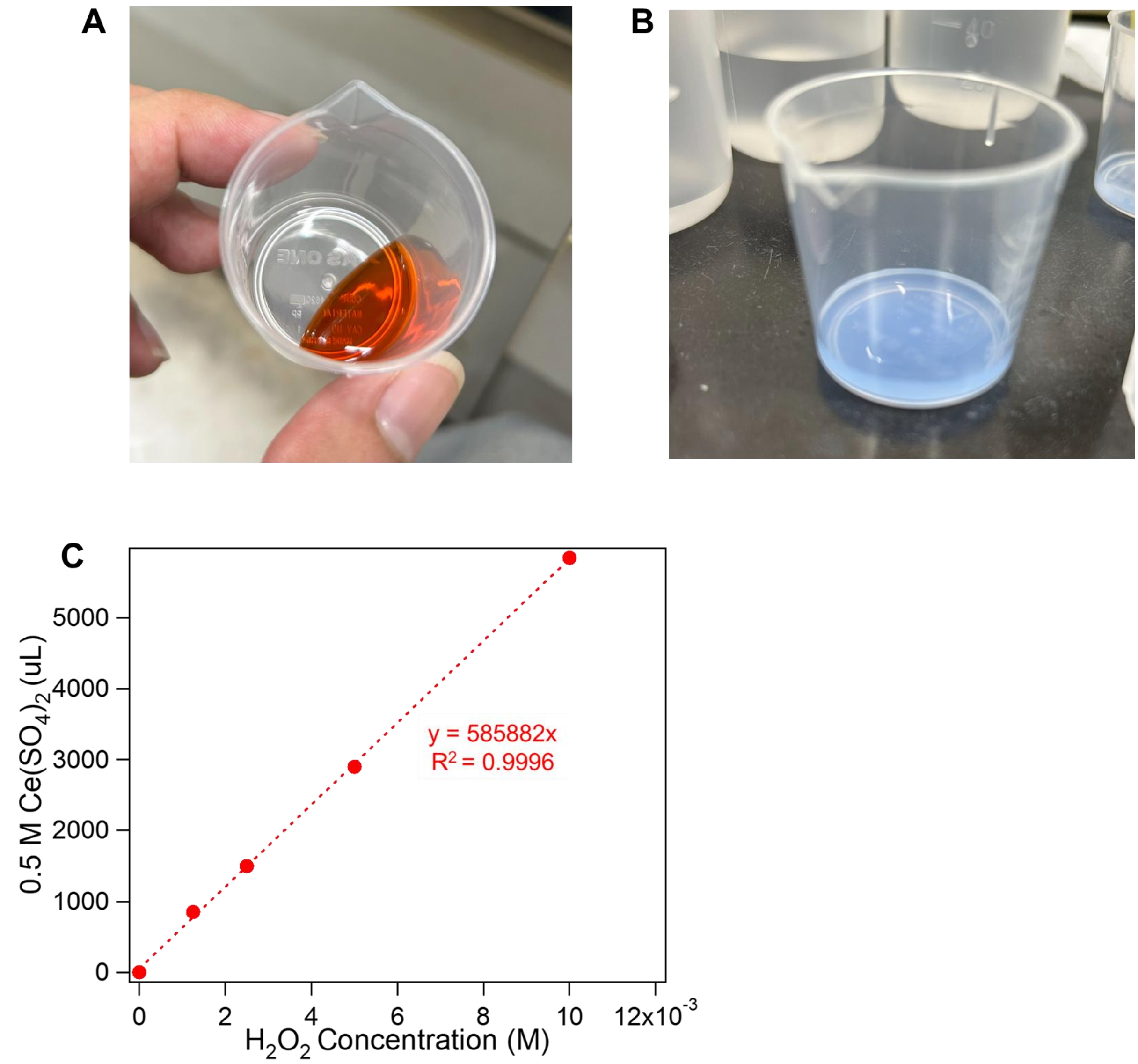

Figure S31. Color of the (A) starting and (B) ending points in the H_2O_2 titration of the post-reaction solution. (C) Calibration curve.

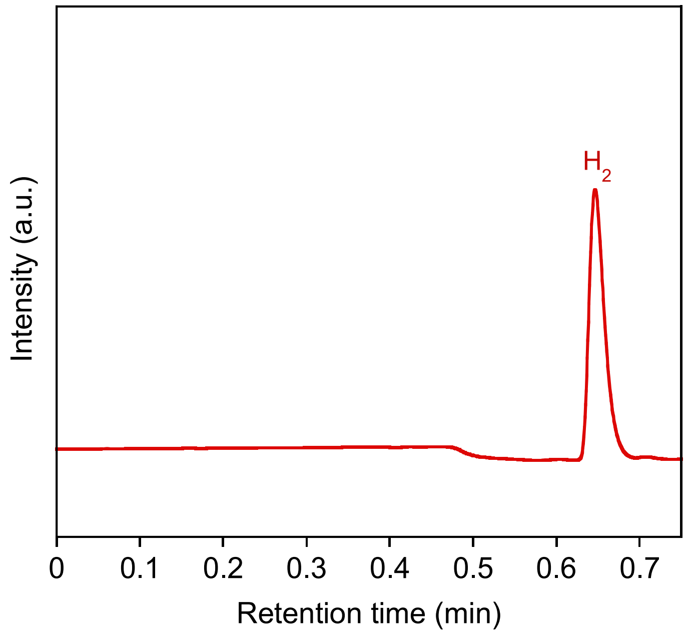

Figure S32. A GC-BID profile of the gas in the reactor after the reaction with CO and solvent (water). Reaction conditions: solvent, 15 mL of H_2O; reaction gas, 5 bar CO; catalyst loading, 5 mg; reaction temperature, 150 °C; reaction time, 1 h. Ar, O_2, CO, C_2H_6, and N_2 appear at > 0.8 min in the present measurement conditions.

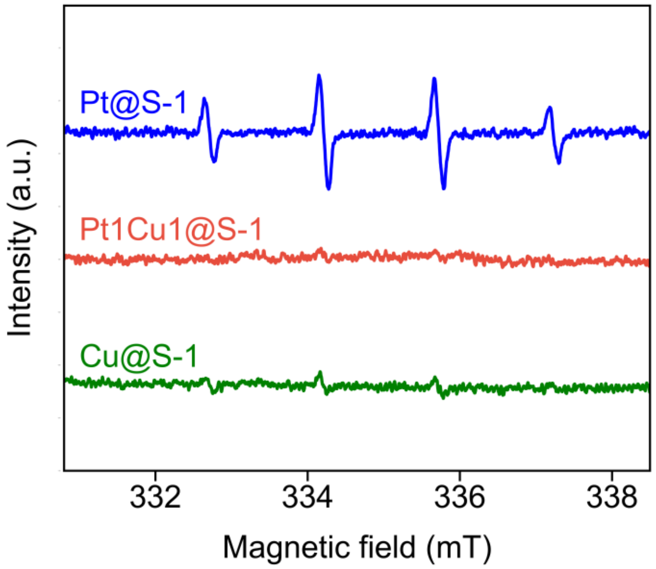

Figure S33. ESR spectra for the post-reaction solution. Reaction conditions: catalyst loading, 50 mg; solvent, 15 ml of 10 mM DMPO aq.; reaction gas, 20 bar CH_4 + 5 bar CO + 3 bar O_2; temperature, 100 ˚C; reaction time, 1 h.

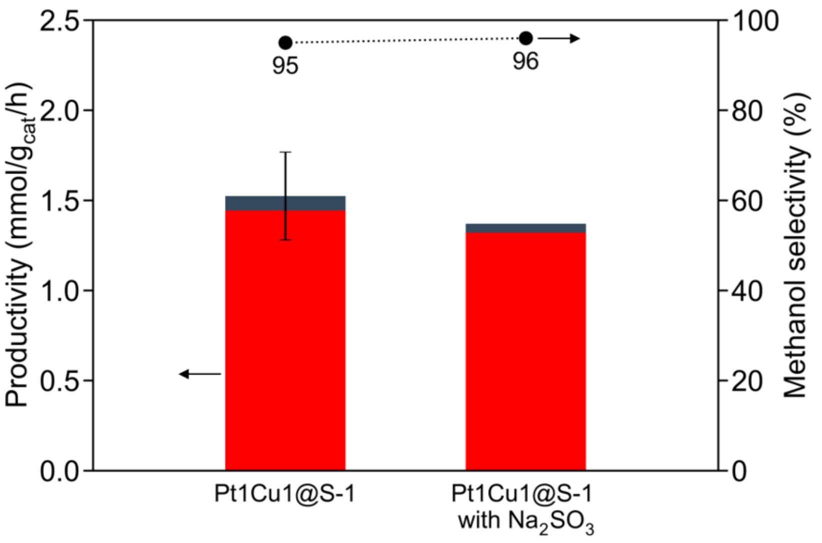

Figure S34. Oxygenates productivity and CH_3OH selectivity in the reaction over Pt1Cu1@S-1 with and without Na_2SO_3. Reaction conditions: solvent, 15 mL of H_2O or 15 mL of 10 mM Na_2SO_3 aq.; reaction gas, 20 bar CH_4 + 5 bar CO + 3 bar O_2; catalyst loading, 5 mg; reaction temperature, 150 °C; reaction time, 1 h.

Table S1. Catalytic performance of previously reported catalysts.[4–11] For direct comparison with this study, only CO-assisted CH_4 oxidation systems are included.

Entry	Catalyst	Temp. [°C]	Gas	Methanol productivity ($mol_{methanol}/mol_{NM}/h$)	$S_{methanol}$ (%)	Ref.
1	Au/HMOR (Si/Al= 12.5, 0.47 wt%)	150	$CH_4/CO/O_2$ (20/5/5 bar)	51.1	94.1	4
2	$1.39Ir_1Cu_1Pd_{0.1}ZSM5$	150	$CH_4/CO/O_2$ (20/5/4 bar)	20.6 (10.8)[a]	93.6	5
3	Rh/MFI	150	$CH_4/CO/O_2$ (20/5/2 bar)	8.5	15.1	6
4	Rh-Na/MFI	150	$CH_4/CO/O_2$ (20/5/2 bar)	3.0	55.4	6
5	Rh-Na/MFI + Cu^{2+}	150	$CH_4/CO/O_2$ (20/5/2 bar)	11.3 (3.8)[a]	84.7	6
6	Rh/TiO_2	150	$CH_4/CO/O_2$ (20/5/2 bar)	1.3	100	6
7	Rh/MFI	150	$CH_4/CO/O_2$ (10/5/2 bar)	51.5	6.2	7
8	Rh/TiO_2 + Cu^{2+}	150	$CH_4/CO/O_2$ (23/5/3 bar)	68.0 (0.2)[a]	100	8
9	H_2-treated Ir	150	$CH_4/CO/O_2$ (15/3/4 bar)	16.4	60.8	9
10	Rh/AEI	150	$CH_4/CO/O_2$ (20/5/2 bar)	5.5	81.6	10
11	0.1 wt% Pt/TiO_2	150	$CH_4/CO/O_2$ (20/5/6 bar)	30.4	62.3	11
12	0.2 wt% Pt/TiO_2	150	$CH_4/CO/O_2$ (20/5/6 bar)	12.4	73.9	11
13	0.5 wt% Pt/TiO_2	150	$CH_4/CO/O_2$ (20/5/6 bar)	0.4	95.5	11
14	Rh/HMOR	150	$CH_4/CO/O_2$ (20/5/5 bar)	11.0	46.2	4
15	Pd/HMOR	150	$CH_4/CO/O_2$ (20/5/5 bar)	5.0	17	4
16	Ir/HMOR	150	$CH_4/CO/O_2$ (20/5/5 bar)	0.9	2.5	4
17	Au/H-MOR 0.07 wt%	150	$CH_4/CO/O_2$ (20/5/5 bar)	72.9	92.8	4
18	Au/H-MOR 0.16 wt%	150	$CH_4/CO/O_2$ (20/5/5 bar)	70.3	96.1	4
19	Au/H-MOR 0.66 wt%	150	$CH_4/CO/O_2$ (20/5/5 bar)	33.8	96.1	4
20	Au/H-MOR 0.87 wt%	150	$CH_4/CO/O_2$ (20/5/5 bar)	23.1	94.8	4
21	Au/H-ZSM-5	150	$CH_4/CO/O_2$ (20/5/5 bar)	28.8	93.4	4
22	Au/H-SSZ-13	150	$CH_4/CO/O_2$ (20/5/5 bar)	27.0	99.7	4

23	Au/H-BEA	150	$CH_4/CO/O_2$ (20/5/5 bar)	22.6	90.7	4
24	Au/SiO_2	150	$CH_4/CO/O_2$ (20/5/5 bar)	10.8	94	4
25	Au/SiO_2 + H-MOR (Si/Al = 12.5)	150	$CH_4/CO/O_2$ (20/5/5 bar)	11.5	91.2	4
26	Au/H-MOR (Si/Al = 20)	150	$CH_4/CO/O_2$ (20/5/5 bar)	16.8	94.7	4
27	Au/H-MOR (Si/Al = 16)	150	$CH_4/CO/O_2$ (20/5/5 bar)	30.8	92.9	4
28	0.1% Pt/TiO_2	150	$CH_4/CO/O_2$ (20/5/6 bar)	37.1	73.9	11
29	0.2% Pt/TiO_2	150	$CH_4/CO/O_2$ (20/5/6 bar))	1.2	95.5	11
30	1% Pt/TiO_2	150	$CH_4/CO/O_2$ (20/5/6 bar))	0	0	11

[a] Methanol productivity ($mol_{methanol}/mol_{total}/h$) which was obtained by methanol formation rate (mol/h) with total moles of noble metal and base metal.

Table S2. Chemical composition of mother zeolite gel for synthesis of metal encapsulated zeolites used in the present study. Amounts of tetraethyl orthosilicate (8.32 g), tetrapropylammonium hydroxide (14 g), water (13.5 g) were constant. The concentrations of noble metal (M_{NM}) and base metal (M_{BM}) ethylenediamine complex aqueous solution were constant (0.045 M).

Catalyst	Al isopropoxide (mg)	M_{NM} ethylenediamine complex aqueous solution (mL)	M_{BM} ethylenediamine complex aqueous solution (mL)
S-1	0	0	0
Pt@S-1	0	2.02	0
Pd@S-1	0	2.02	0
Ir@S-1	0	2.02	0
Rh@S-1	0	2.02	0
Pt3Cu1@S-1	0	1.51	0.52
Pt1Cu1@S-1	0	1.01	1.01
Pt1Cu3@S-1	0	0.52	1.51
Pt1Co1@ S-1	0	1.01	1.01
Pt1Ni1@ S-1	0	1.01	1.01
Rh1Cu1@S-1	0	1.01	1.01
Pd1Cu1@S-1	0	1.01	1.01
Ir1Cu1@S-1	0	1.01	1.01
Pt3Cu1@MFI (Si/Al=274)	237	1.51	0.52
Pt3Cu1@MFI (Si/Al=181)	316	1.51	0.52
Pt3Cu1@MFI (Si/Al=127)	553	1.51	0.52

Table S3 Textural properties and chemical compositions. All chemical compositions were determined by ICP-OES.

Catalyst	Pt loading (wt%)	Cu loading (wt%)	Cu/Pt molar ratio	Si/Al molar ratio	S_{BET} (m^2/g)	V (cm^3/g)
Pt@S-1	0.48	-	∞	∞	441	0.25
Pt3Cu1@S-1	0.32	0.07	0.67	∞	446	0.26
Pt1Cu1@S-1	0.21	0.13	1.84	∞	427	0.22
Pt1Cu3@S-1	0.10	0.19	5.92	∞	435	0.24
Cu@S-1	-	0.26	-	∞	412	0.37
Pt1Cu1/S-1	0.21	0.13	1.84	∞	453	0.27
Pt3Cu1@MFI(274)	0.30	0.09	0.97	274	437	0.23
Pt3Cu1@MFI(181)	0.28	0.09	1.02	181	437	0.24
Pt3Cu1@MFI(127)	0.24	0.09	1.11	127	442	0.24
S-1	-	-	-	∞	441	0.24

Table S4. Best fit EXAFS parameters at the Pt L_{III}-edge of Pt1Cu1@S-1.

Ref.	Backscattering	R (Å)	CN	σ^2(Å^2)
Pt1Cu1@S-1 fresh	Pt–Pt	2.71±0.002	5.5±0.4	0.006±0.0003
	Pt–Cu	2.64±0.005	4.1±0.4	0.012±0.0007
[12]	Pt–Pt	2.71	4.6	0.004
	Pt–Cu	2.64	4.5	0.016
[13]	Pt–Pt	2.71±0.03	7.2±0.7	0.0049±0.0005
	Pt–Cu	2.67±0.01	4.1±0.8	0.0091±0.0016

Table S5. Catalytic performances of PtCu@S-1 catalysts with different Cu/(Cu+Pt) molar ratios.[a]

Catalyst	Productivity ($mmol/g_{cat}/h$)				Methanol productivity ($mol_{methanol}/mol_{Pt}/h$)	$S_{methanol}$ (%)
	CH_3OH	CH_3OOH	HCOOH	CH_3COOH		
Pt@S-1	0.10	0.08	0.15	0.00	4.2	31
Pt3Cu1@S-1	1.30	0.03	0.00	0.05	79.0 (47.3)[b]	94
Pt1Cu1@S-1	1.44	0.00	0.00	0.08	134.0 (46.2)[b]	95
Pt1Cu3@S-1	0.50	0.02	0.00	0.05	97.9 (14.3)[b]	87
Cu@S-1	0.00	0.00	0.00	0.00	0.0	0

[a] Reaction conditions: catalyst, 5 mg; solvent, 15 ml of H_2O; reaction gas, 20 bar CH_4 + 5 bar CO + 3 bar O_2; temperature, 150 ˚C; reaction time, 1 h.

[b] Methanol productivity ($mol_{methanol}/mol_{total}/h$) which was obtained by methanol formation rate (mol/h) with total moles of Pt and Cu.

References

(1) Ravel, B.; Newville, M. ATHENA and ARTEMIS: Interactive Graphical Data Analysis Using IFEFFIT. *Phys. Scr.* **2005**, *2005* (T115), 1007.

(2) Funke, H.; Scheinost, A. C.; Chukalina, M. Wavelet Analysis of Extended X-Ray Absorption Fine Structure Data. *Phys. Rev. B.* **2005,** *71*, 094110.

(3) Funke, H.; Chukalina, M.; Scheinost, A. C. A New FEFF-Based Wavelet for EXAFS Data Analysis. *J. Synchrotron Rad.* **2007**, *14*, 426–432.

(4) Wang, W.; Zhou, W.; Tang, Y.; Cao, W.; Docherty, S. R.; Wu, F.; Cheng, K.; Zhang, Q.; Copéret, C.; Wang, Y. Selective Oxidation of Methane to Methanol over Au/H-MOR. *J. Am. Chem. Soc.* **2023**, *145*, 12928–12934.

(5) Li, M.; Shan, J.; Giannakakis, G.; Ouyang, M.; Cao, S.; Lee, S.; Allard, L. F.; Flytzani-Stephanopoulos, M. Single-Step Selective Oxidation of Methane to Methanol in the Aqueous Phase on Iridium-Based Catalysts. *Appl. Catal. B* **2021**, *292*, No. 120124.

(6) Shan, J.; Li, M.; Allard, L.; Lee, S.; Flytzani-Stephanopoulos, M. Mild Oxidation of Methane to Methanol or Acetic Acid on Supported Isolated Rhodium Catalysts. *Nature* **2017**, *551*, 605–608.

(7) Tang, Y.; Li, Y.; Fung, V.; Jiang, D.-E.; Huang, W.; Zhang, S.; Iwasawa, Y.; Sakata, T.; Nguyen, L.; Zhang, X.; Frenkel, A. I.; Tao, F. F. Single Rhodium Atoms Anchored in Micropores for Efficient Transformation of Methane under Mild Conditions. *Nat. Commun.* **2018**, *9*, 1231.

(8) Gu, F.; Qin, X.; Li, M.; Xu, Y.; Hong, S.; Ouyang, M.; Giannakakis, G.; Cao, S.; Peng, M.; Xie, J.; Wang, M.; Han, D.; Xiao, D.; Wang, X.; Wang, Z.; Ma, D. Selective Catalytic Oxidation of Methane to Methanol in Aqueous Medium over Copper Cations Promoted by Atomically Dispersed Rhodium on TiO_2. *Angew. Chem., Int. Ed.* **2022**, *61*, No. e202201540.

(9) Li, H.; Fei, M.; Troiano, J. L.; Ma, L.; Yan, X.; Tieu, P.; Yuan, Y.; Zhang, Y.; Liu, T.; Pan, X.; Brudvig, G. W.; Wang, D. Selective Methane Oxidation by Heterogenized Iridium Catalysts. *J. Am. Chem. Soc.* **2023**, *145*, 769–773.

(10) Moteki, T.; Tominaga, N.; Tsunoji, N.; Yokoi, T.; Ogura, M. Impact of the Zeolite Cage Structure on Product Selectivity in CO-Assisted Direct Partial Oxidation of Methane over Rh Supported AEI-, CHA-, and AFX-Type Zeolites. *Chem. Lett.* **2021**, *50*, 1597–1600.

(11) Yin, H.; Wu, B.; Ma, X.; Su, G.; Han, M.; Lin, H.; Liu, X.; Li, H.; Zeng, J. CO-Assisted Methane Oxidation into Oxygenates over Surface Platinum-Titanium Alloyed Layers. *Nano Lett.* **2024,** *24*, 5002–5009.

(12) Deng, Z.; Gong, Z.; Gong, M.; Wang, X. Multiscale Regulation of Ordered PtCu Intermetallic Electrocatalyst for Highly Durable Oxygen Reduction Reaction. *Nano Lett.* **2024**, *24*, 3994–4001.

(13) Tahsini, N.; Yang, A.-C.; Streibel, V.; Werghi, B.; Goodman, E. D.; Aitbekova, A.; Bare, S. R.; Li, Y.; Abild-Pedersen, F.; Cargnello, M. Colloidal Platinum-Copper Nanocrystal Alloy Catalysts Surpass Platinum in Low-Temperature Propene Combustion. *J. Am. Chem. Soc.* **2022**, *144*, 1612–1621.
